# Medicinal Plants for Treating Musculoskeletal Disorders among Karen in Thailand

**DOI:** 10.3390/plants9070811

**Published:** 2020-06-28

**Authors:** Rapeeporn Kantasrila, Hataichanok Pandith, Henrik Balslev, Prasit Wangpakapattanawong, Prateep Panyadee, Angkhana Inta

**Affiliations:** 1 Department of Biology, Faculty of Science, Chiang Mai University, Chiang Mai 50200, Thailand; rapeeporn_ka@cmu.ac.th (R.K.); hataichanok.p@cmu.ac.th (H.P.); prasit.wang@cmu.ac.th (P.W.); 2 Department of Biology, Aarhus University, Building 1540, Ny Munkegade 116, DK-8000 Aarhus C, Denmark; henrik.balslev@bios.au.dk; 3 Queen Sirikit Botanic Garden, the Botanical Garden Organization, Chiang Mai 50180, Thailand; prateep@qsbg.mail.go.th; 4 Research Center in Bioresources for Agriculture, Industry and Medicine, Chiang Mai University, Chiang Mai 50200, Thailand

**Keywords:** ethnobotany, MSD, Pwa Ka Nyaw, traditional knowledge

## Abstract

Millions of people suffer from Musculoskeletal System Disorders (MSDs), including Karen people who work hard in the fields for their subsistence and have done so for generations. This has forced the Karen to use many medicinal plants to treat MSDs. We gathered data from 15 original references covering 27 Karen communities and we document 461 reports of the use of 175 species for treating MSDs among the Karen people in Thailand. The data were analyzed by calculating use values (UV), relative frequency of citation (RFC) and informant consensus factor (ICF). Many use reports and species were from Leguminosae and Zingiberaceae. Roots and leaves were the most used parts, while the preferred preparation methods were decoction and burning. Oral ingestion was the most common form of administration. The most common ailment was muscle pain. *Sambucus javanica* and *Plantago major* were the most important species because they had the highest and second-highest values for both UV and RFC, respectively. This study revealed that the Karen people in Thailand use various medicinal plants to treat MSDs. These are the main resources for the further development of inexpensive treatments of MSDs that would benefit not only the Karen, but all people who suffer from MSD.

## 1. Introduction

Traditional knowledge of medicinal plants is transferred from generation to generation in local communities [1]. Plants are used over a lifetime from birth to death [2]. Although modern medicines are much used everywhere around the world, traditional medicines are still important to many people, especially among ethnic minority groups [3,4] and in developing countries [5,6,7,8]. For example, a high proportion of the population in Africa, Chile, and Pakistan, still rely on traditional medicine [9,10]. The uses of medicinal plants are still popular because they are inexpensive, easy to use, and they have limited side effects compared to modern medicines [11].

Musculoskeletal disorders (MSDs) are non-communicable diseases and they are dramatically increasing in many developing and developed countries [12]. More than 1.7 billion people throughout the world suffer from these ailments, causing both disability and death [13]. Recently, the World Health Organization (WHO) reported that MSDs, such as osteoarthritis, arthritis, back and neck pain, and bone fractures, are the second most common cause of disability in the world [14]. These disorders do not only occur among the elderly, but also hit adolescent people because they work hard throughout life. About 20–33% of the world’s population have experienced painful and disabling muscular-skeleton conditions. In the USA, one of two adults have suffered from such ailments [14]. In Europe, MSDs are one of the most common causes of severe long-term pains and disabilities, leading to significant healthcare and social support costs [15]. In addition, limited mobility, and adroitness, caused by MSDs, can lead to the loss of work and reduced capability in social roles [14]. In Asia, there is a high prevalence of arthritis in all countries, but especially in India and China [16].

People from many parts of the world have used a number of medicinal plants for treating ailments related to MSDs, such as muscular pain, rheumatism, fractured bones, etc. Studies in Turkey [17] and Pakistan [10] listed 142 plant species, which were traditionally used to treat MSDs, mostly rheumatism. Moreover, professional farmers are much affected by MSDs. For example, farmers in southeast Kansas (USA) [18], the Netherlands [19], Britain, and Ireland [20] were reported to suffer injuries from MSDs. Important ailments of MSDs included osteoarthritis, lower back pain, upper limb disorders, sprains, fractures, and dislocations [21].

In Thailand, the consequences of MSDs are severe. Thailand is an agricultural country in which rice farming occupies over half of the total agricultural area [22]. Farmers’ physical activities include excessive bending, twisting, kneeling, and carrying loads, which have caused many ailments related to MSDs [12,23,24,25]. These ailments commonly affect the lower back, shoulders, hands/wrists and knees [26,27]. However, even if Thailand has been the subject of many ethnomedicinal studies, none of them have focused on medicinal plants to treat MSDs (e.g., Kantasrila [28] and Kaewsangsai [29]).

Here, we studied the Karen, who are the largest ethnic minority group in Thailand. The Karen people live, mostly, in the Tak, Mae Hong Son, Chiang Mai, Ratchaburi, and Kanchanaburi provinces. Most of them settle in the mountainous areas above 500 m above sea level. Their livelihoods are based on agriculture [28,29,30] and they cultivate rice in swidden fields around their villages using only a few agricultural machines [28,31]. They spend a long time bending their body which, in turn, produces a high risk of back injury, muscular pain, and fatigue from farming. Treatments in hospitals, which are often located far away from their villages, take a long time and cost both time and money [28]. Thus, most rural farmers use traditional treatments that involve many medicinal plants to cure their ailments.

Accordingly, it is important to document ethnobotanical information among the Karen to find: (1) How many species of plants are used to treat MSDs? (2) What are the most important plant species and families used for treating MSDs? (3) What are the preferred plant parts and methods of preparation of plants for treating MSDs? (4) Which of the MSD categories has the highest prevalence among the Karen and which plants are used to treat them? The outcome of this research could facilitate the identification and selection of plant species as effective treatments for MSD patients.

## 2. Results

### 2.1. Medicinal Plant Diversity

**Figure 1:**
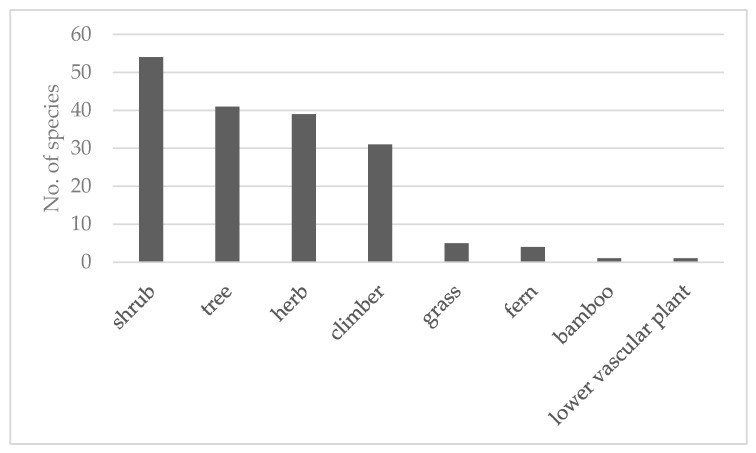
Habit of the medicinal plants used to treat MSDs among the Karen in Thailand.

A total of 461 use reports were compiled from 15 references that covered 27 villages from the Chiang Mai and Mae Hong Son provinces in northern Thailand and the Kanchanaburi, Ratchaburi, and Tak provinces in western Thailand. The use reports related to 175 species in 144 genera and 75 families, as shown in Table 1 and Table S1. Most of them (170 spp.) were flowering plants, including 53 species of shrubs, 41 species of trees, 39 species of herbs, 31 species of climbers, 5species of grass and 1 species of bamboo, as shown in Figure 1. The families with most species of MSD medicinal plants were Leguminosae (12 species, 31 use reports), Zingiberaceae (10 species, 19 use reports), Rubiaceae (9 species, 10 use reports), and Asteraceae (8 species, 36 use reports).

### 2.2. Plant Part Used, Preparation and Routes of Administration

**Figure 2:**
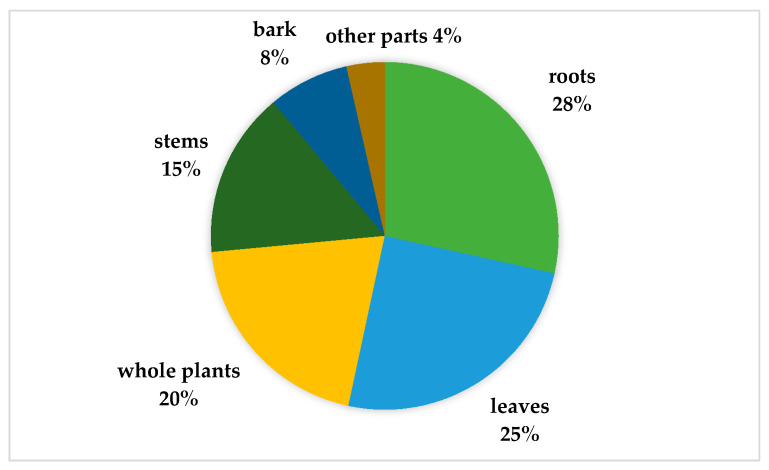
Plant parts used to treat MSDs among Karen communities in Thailand.

In terms of plant parts used, they were significantly different between the use reports of each part (Chi-square test, *p* < 0.05). The root was the most used part for treating MSDs. It was mentioned in 28% of all use reports, followed by leaves (25%) and whole plants (20%), respectively, as shown in Figure 2.

**Figure 3:**
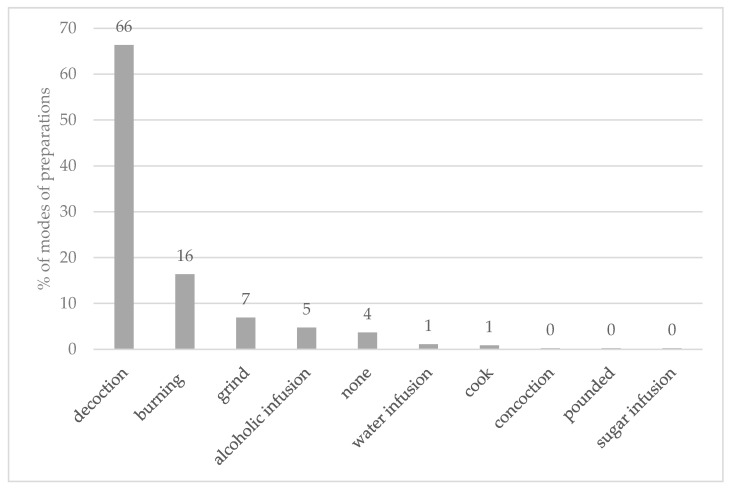
Modes of preparation of medicinal plants used to treat MSD among the Karen in Table 2. Musculoskeletal Disorders Categories.

Considering the mode of preparation of medicinal plants to treat MSDs, the use reports of preparation were significantly different between the methods (Chi-square test, *p* < 0.05). There were many methods for preparing medicinal plants, as shown in Figure 3. Among these, decoction and burning were most common, contributing 66% and 16%, respectively, of the total use-reports.

Regarding the route of administration, there were diverse ways of using medicinal plants. Oral ingestion was the most preferred method (68%), which was significantly different from the other applications (Chi-square test, *p* < 0.05), followed by poultices (21%). Eaten as food, compress, bath, steaming, chewing, liniment, and soak had low use reports.

**Figure 4:**
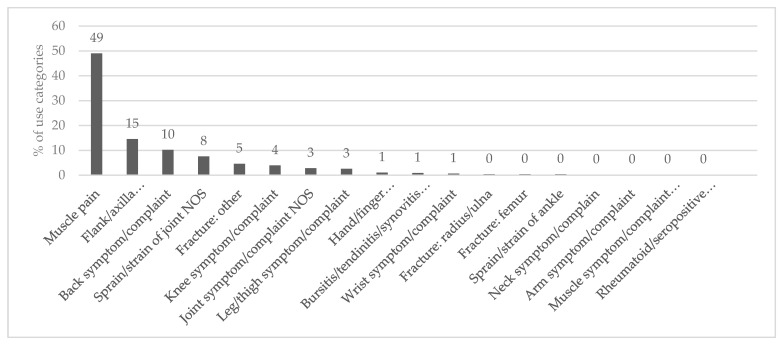
Categories of MSDs treated with medicinal plants among the Karen in Thailand.

The 461 reports belonged to 18 use categories, as shown in Figure 4, according to the International Classification of Primary Care [32]. They were significantly different between the use reports of each category (Chi-square test, *p* < 0.05). The largest category was muscular pain (49%), followed by flank/axilla symptom/complaint (15%) and back symptom/complaint (10%), respectively. In the other extreme, there was only one report for each of the following use categories: neck symptom/complain, arm symptom/complaint, muscle symptom/complaint NOS (Not Otherwise Specified), and rheumatoid/seropositive arthritis.

Sometimes different plants were used to treat the same ailment using the same preparation in different Karen villages. For example, in 16 villages they used the leaves of *Sambucus javanica* Reinw. ex Blume, to treat fractured bones and muscle pains by burning them, then placing them on the painful areas. The leaves of *Plantago major* L. were ground and put on the painful joints. This was reported from ten villages. Many species were reported for their uses in more than one use category. For instance, *Blumea balsamifera* (L.) DC, was used to treat back pains (back symptom/complaint), lumbar pains (flank/axilla symptom/complaint), muscle pains (muscle pain), and sprains (sprain/strain of joint NOS), as shown in Table 1.

### 2.3. Ethnobotanical Indices: UV, RFC, and ICF

#### 2.3.1. Use Values (UV) of the Ethnomedicinal Plants for Treating MSDs

UVs, calculated to compare the importance of the different species of medicinal plants, ranged from 0.037–1.148. Species with high UVs included: *Sambucus javanica* (1.148), *Plantago major* (0.852), *Miliusa thorelii* Finet and Gagnep (0.704), *Pothos scandens* L. (0.630), *Sambucus simpsonii* Rehder (0.481), *Blumea balsamifera* (0.407), and *Duhaldea cappa* (Buch.-Ham. ex D. Don) Pruski and Anderb. (0.407), as shown in Table 1. At the other extreme, a large number of medicinal plants (49%) were cited only once for their uses to treat MSD ailments.

#### 2.3.2. The Relative Frequency of Citations (RFC) of the Ethnomedicinal Plants

The RFC ranged from 0.593–0.037. The plant with the highest RFC value was *Sambucus javanica* (0.593) followed by *Plantago major* (0.370), *Gmelina arborea* Roxb. (0.296), *Duhaldea cappa* (0.259), *Miliusa thorelii* (0.259), *Pothos scandens* (0.259), *Sambucus simpsonii* (0.259), and *Elephantopus scaber* L. (0.222). However, it should be noted that more than half of the medicinal plants used to treat MSDs had low RFC values (RFC = 0.037). These plants were known in only one village, as shown in Table 1.

#### 2.3.3. The Information Consensus Factors (ICF) of MSD Categories

The Information consensus factors (ICF) ranged from 0–0.75, as shown in Table 2. The ailment category with the highest ICF was hand/finger symptom/complaint (0.75), followed by fracture: other (0.67), sprain/strain of joint NOS (not otherwise specified) (0.58), joint symptom/complaint NOS (0.56), bursitis/tendinitis/synovitis NOS (0.50), and wrist symptom/complaint (0.50) categories. On the other hand, there were seven categories with the ICF values equal to zero, including arm symptom/complaint, fracture: femur, fracture: radius/ulna, muscle symptom/complaint, neck symptom/complain, and rheumatoid/seropositive.

## 3. Discussion

### 3.1. Diversity of Medicinal Plant Used to Treat MSD

There was a high diversity of medicinal plants used to treat MSDs among the Karen communities. These plants make up 30% of all medicinal plant species in Thailand, when compared with the review of ethnobotanical knowledge about medicinal plants to treat MSDs in Thailand [33]. This implies that MSDs have a high prevalence among the Karen in Thailand. That may be why they use so many plant species to treat these ailments. It should be noted that the number of medicinal MSD plants is different in different villages. Many villages had a high number of MSD plants. Many medicinal plants were used in only a single village. This shows that the knowledge of plant used to deal with MSDs could originate independently in individual villages. Moreover, knowledge is hard to exchange among different villages because of their isolation.

Leguminosae were the most prominent family for treating MSD among the Thai Karen people, which agrees with other ethnomedicinal research around the world [34,35,36,37]. Leguminosae were reported to have the highest number of medicinal plant species used to treat MSDs in northern Pakistan [10]. Many species of the family are used by local people in different parts of world to cure ailments [38]. Moreover, it was also one of the dominant families in ethnobotanical plant surveys, with the highest number of use reports and used species among several ethnic groups in Thailand [33]. The Karen used many medicinal Leguminosae and still maintain a substantial traditional plant knowledge [39]. Leguminosae is one among the largest plant families globally [40] and it is found in various habitats and attains various life forms. Therefore, it was selected for use in highland regions of southeast Asia [41]. Other plant families with many medicinal plant species were Zingiberaceae, Asteraceae, and Rubiaceae, which also have many species in Thailand [33,42]. Asteraceae is another large family, together with Leguminosae, in terms of global numbers of species [43]. Both families have many species that are used to treat MSD ailments [10]. All these families are also dominant in other ethnobotanical studies in Thailand [33].

Shrubs and trees were the most common life forms of the plants harvested by the Karen people for traditional medicine for MSDs. Trees were especially commonly used for MSD treatments in other parts of the world, such as India [37], Ghana [44], Peru, and South America [45].

### 3.2. Plant Utilization: Parts, Preparation, and Routes of Administration

Leaves and roots were the most used parts in the treatment of MSDs, similar to what has been found in other studies in Thailand, such as the ethnobotany of the Mien (Yao) in northern Thailand [46,47], and the review of all ethnomedicinal uses of plants in Thailand [33]. Leaves were reported as the most used part in several other ethnomedicinal studies of MSD treatments around the world, such as in Algeria [48], Central Africa [49,50], India [37], Italy [51], Kenya [52], Papua New Guinea [53], and South Africa [54]. Additionally, leaves and roots were greatly used for the treatment of MSDs in northern Pakistan [10]. Leaves are often preferred because they can be harvest easier than other parts of the plant [46,55]. Moreover, leaves are rich in secondary metabolites because they are the site of photosynthesis [49,56]. Another much used part was the root because some bioactive compounds are preserved in roots in higher concentrations than in other parts [57].

The most used method of preparation was decoction. This method is common for preparing medicinal plants in Thailand [33,58] and around the world, such as in Central Africa [59], China [60], eastern Nicaragua [61], northern Pakistan [10], and the Philippines [35]. Decoction is the easiest way to extract bioactive substances from plant materials [33]. Moreover, sweeteners, such as sugar or honey, can be added to the decoction during or after the preparation to adjust the taste and reduce the bitterness of the medicines [33,62,63]. Besides drinking, the decoction could also be applied externally (e.g., in bathing) [64].

The preferred route of administration was oral ingestion. It was reported to be the most common method of administration in other studies in Thailand [46,47] and many areas around the world, such as India [37] and Papua New Guinea [65,66]. Other favored routes of administration were poultices and eaten as food. Medicinal plants were prepared by grinding and applied directly to the injured parts. In addition, when the plants were crushed or ground, they released their secondary compounds [67,68]. Additionally, eating vegetables as food made patients feel like they did not take any medicine [33]. Medicinal plants, which were prepared as food, could be eaten as fresh vegetables, which is an easy way to prepare them because they can be eaten as a part of the daily diet [64].

### 3.3. Important Disorder Categories

Most species were used to treat ailments in the muscular pain category. This result was similar to reports from other areas, such as northern Pakistan [10] and Spain [69]. The muscular pain category was a dominant MSD category, and many communities around the world have used many medicinal plants to treat it [70]. Famers have used many medicinal plants to treat muscle pain caused by laborious work in the fields [71]. They spend a lot of time cultivating rice without the help of agricultural machines, which may cause muscle pain. In addition, many medicinal plants were used to treat flank/axilla symptom/complaint and back symptom/complaint. According to previous research, the most prevalent MSD in farmers was pain in the lower back due to physical activities, such as excessive bending, twisting, and carrying of loads [12]. Moreover, these activities commonly affected other parts of the body, such as the shoulders, hands/wrists, and knees among the farmers [12,23,24,25,26,27].

### 3.4. Important Plants for Treating MSD

#### 3.4.1. Most Preferred Species for Treating MSD

The UVs depend on use reports and the commonness of plants around the studied areas. Plant species with high UV values indicated that they had use reports and were commonly found in the studied areas [33,72]. UV could be calculated to show which species were important to the communities, while RFC determined the level of traditional knowledge about the use of medicinal plants in the study areas. When the RFC values were high, it referred to common popularity, utilization, and priority species among informants for curing specific ailments [10]. The most important plant for treating MSDs among the Karen people was *Sambucus javanica*. It had both high UVs and RFC values. It was used in many categories of MSD (e.g., flank/axilla symptom/complaint, fracture, joint symptom/complaint, leg/thigh symptom/complaint, muscle pain, sprain/strain of joint and wrist symptom/complaint). Moreover, it was reported in 16 (60%) of the 27 villages for which we had data. This plant is well known for its medicinal properties among villagers of many other ethnic groups in Thailand. It is used for treating bone fractures and muscle pain by the Akha [58,73], the Hmong [74], the Karen [58], the Lua [74], the Mien [58,74], and the Thai Yuan communities [74]. Another species in the same genus, *Sambucus simpsonii*, also had high UVs and RFC values. This plant is the cultivated version of *S. javanica* and it was used as a substitute for *S. javanica*. Other species in this genus have been reported to have phytochemical contents with anti-inflammatory and anti-analgesic properties, which may be directly related to their use for treating MSDs. One example is *Sambucus williamsii* Hance, which is used to treat bone and joint diseases in China [75]. It has compounds, such as phenolics and terpenoids, which have anti-inflammatory effects [75]. The root extract of *Sambucus ebulus* L., also had anti-inflammatory and anti-analgesic effects [76]. Elderberry, *Sambucus nigra* L., is known for its phenolics and flavonoids with similar antioxidant activity [77].

Other species with high UV and RFC values were *Plantago major*, *Miliusa thorelii*, *Pothos scandens*, *Gmelina arborea*, *Elephantopus scaber*, *Duhaldea cappa*, and *Blumea balsamifera*. These species were reported in many Karen villages and were used to treat ailments in many MSD categories. Some of them are cosmopolitan, such as *Plantago major*, and they are easy to collect for use. This plant was reported as being used in eight MSD categories, such as back symptom/complaint, flank/axilla symptom/complaint, muscle pain, etc. It contains iridoids with relenting anti-inflammatory activity that could relieve MSD [78]. Many ethnic groups, including Karen [58], Tai-Yai [79], Mien [58,79] Akha [58], and Hmong [58], also used it to treat rheumatic ailments, bone fractures, and muscle pains [58,78,79]. *Blumea balsamifera* has been used for traditional medicine for thousands of years in Southeast Asia [80]. Moreover, this plant has chemical compounds with anti-inflammatory [81] and antioxidant effects [80,82].

*Gmelina arborea* [83,84], *Elephantopus scaber* [85,86], and *Duhaldea cappa* [87], were also used for their anti-inflammatory properties. For instance, *Gmelina arborea* [84] and *Elephantopus scaber* [85,86] have flavonoids, tannins, and saponins. *Miliusa thorelii* and *Pothos scandens* have been used for curing many MSD categories in this study, such as fractures, joint symptoms, and muscle pains, but any phytochemicals that could affect MSD remain to be documented in these species.

#### 3.4.2. Important Species in Important Disorders

High ICF values indicate a high level of agreement between informants in terms of using medicinal plants to treat diseases [88]. In addition, high ICF values are important for selecting plants for studies of their bioactive compounds [89]. However, the values of ICF should be considered, together with the number of use reports. Categories with low numbers of use reports could give rise to unusually high ICF values. For example, the category, hand/finger symptom/complaint, had the highest ICF value, 0.75. However, only five use reports from two species were recorded for this category, including *Curcuma elata* Roxb. and *Plantago major.* Other categories also had high ICF values, including fracture: other, sprain/strain of joint, joint symptom/complaint, bursitis/tendinitis/synovitis, and wrist symptom/complaint. The Fracture: other category had the second highest ICF value, but it had few citations and plant species. The most popular species in this group were *Sambucus javanica* and *Sambucus simpsonii*. Both categories, sprain/strain of joint and joint symptom/complaint, had relatively few use reports and species when compared with muscle pain categories, which had the highest use value and number of species. However, considering the use reports of these groups, it appears that the informants had similar knowledge about plant uses. The species which were the most popular among informants in sprain/strain of joint and joint symptom/complaint were *Sambucus javanica* (27% of total use report) and *Plantago major* (13% of total use report), respectively. On the other hand, bursitis/tendinitis/synovitis and wrist symptom/complaint had very low numbers of both the use reports and the species. There were two species with three use reports. Some medicinal plants were reported to treat bursitis/tendinitis/synovitis and wrist symptom/complaint, including *Flacourtia rukam* Zoll. and Moritzi, *Bistorta paleacea* (Wall. ex Hook.f.) Yonek. and H. Ohashi, *Sambucus javanica* and *Tupistra muricata* (Gagnep.) N. Tanaka, respectively. This implies that these categories were not prevalent among the informants.

The muscle pain category had the highest numbers of citations and species used. The ICF value of this group was 0.38, demonstrating a great diversity in the knowledge of medicinal plants for the treatment of ailments in the muscle pain category. The most popular species in this group were *Blumea balsamifera* and *Sambucus javanica*, both with high values for use values.

## 4. Materials and Methods 

### 4.1. Data Source

The data about medicinal plants used for treating MSD by the Karen in Thailand were compiled from 15 ethnobotanical references, which included unpublished scientific reports and published journal articles, as shown in Table 3. The references were produced in the period 1995–2017. They were extracted from online theses of the Thai Library Integrated System, which cover all theses of Thai universities. Some additional data were extracted from theses and un-published research reports of the Ethnobotany and Northern Thai Flora Laboratory, Department of Biology, Chiang Mai University. In order to avoid data duplication, we followed the procedure proposed by Phumthum et al. [33] by excluding research articles and duplicated research studies by the same authors and study areas. In total, 27 Karen villages were covered by the data in this review, including 21 villages in the Chiang Mai province, two villages in the Mae Hong Son and Ratchaburi provinces, and one village in each of the Tak and Kanchanaburi provinces.

### 4.2. Data Organization

The scientific species and family names of the medicinal plants were verified following Plants of The World Online and Flora of Thailand. Plant use data were classified into medicinal categories of MSDs following the International Classification of Primary Care, Second edition (ICPC-2) [32]. The ICPC-2 classification system is based on body system. The disorders were classified according to specific body systems or to non-specific categories: not otherwise specified (NOS). For example, the muscle pain category included specific sub-categories, such as fibromyalgia, fibrositis, myalgia, panniculitis, and rheumatism, whereas other disorders involving the muscles of the body were classified into muscle system/complaint NOS categories. The vernacular names were as mentioned in the references. The parts of the plants used were derived from the references and classified into: roots, leaves, stem, bark, inflorescences, infructescence, whole plants, aerial parts, and not specified. Methods of preparation and routes of administration followed the original reports.

### 4.3. Data Analysis

The ethnobotanical knowledge was collected as “use report”. Each “use report” refers to the use of a specific species with a specific method of preparation, which was used to treat an ailment in an MSD category in a Karen village. Because this is a meta-analysis where we only knew the village studied and not the individual informants interviewed, we used the village as a “pseudoinformant” in our analysis. The pseudoinformant was a representative of traditional knowledge about the medicinal plant usage of each village. It showed all medicinal plant species to treat the MSDs of each village. Therefore, if the data reported that a species was used to treat the same MSD category, but it had different methods of preparation, then each method was counted as a separate use report. For example, if species A was boiled for drinking or burned for a body compress to treat muscle pain, then these were counted as two use reports. The significant differences of use reports among different categories were analyzed by a Chi-square test with α = 0.05. This analysis was performed by SPSS software, version 17. The Chi-square test was performed to test significant difference among the studied variables of use reports with α = 0.05. Moreover, ethnobotanical indices were used in order to find the important and preferred medicinal plants for treating MSD among the Karen. These methods were modified from Phumthum et al. [33].

#### 4.3.1. Use Value (UV) Modified from:

UV = (∑Ui)/N

where Ui is the number of use-reports mentioned by each pseudoinformant and N refers to the total number of pseudoinformants [104]. For example, when the original reference recorded the use of a plant from three different villages, this would count as three use reports from three pseudoinformants.

Use values are high when there are many use reports for a plant, implying that the plant is important, and in contrast, UVs approach zero when there are few reports related to its use [105].

#### 4.3.2. Relative Frequency of Citation (RFC)

This index showed the local importance of each plant used among the informants. It was calculated as:

RFC = FC/N

where FC is the number of pseudoinformants who mention the use of the species and N is the total number of pseudoinformants who participated in the study (27).

The value of RFC ranges from 0 to 1. When RFC is 0, it means no informant use the species in question. On the other hand, RFC is equal to 1 when all informants mention the use of the species [106].

#### 4.3.3. Informant Consensus Factor (ICF)

This index was used to analyze the rank of agreement among informants for medicinal plants used in each category [107]. The ICF was calculated as:

ICF = (Nur − Nt)/(Nur − 1)

where Nur refers to the number of use reports for a particular use category and Nt refers to the number of taxa recorded in that same category. ICF is low (near 0) when most informants report different plants for a category. This would imply that plants were chosen randomly for use in that category or no exchange of information had occurred about the medicinal plants used among informants. However, the ICF value is high (approaching 1) when a few plants are reported by a high proportion of informants for the same use, also implying that the exchange of knowledge had occurred between informants [108].

## 5. Conclusions

Our review compiles ethnobotanical knowledge of the Karen people about plants used to treat musculoskeletal disorders. We found 175 medicinal plant species belonging to 144 genera and 75 families. The most important species were *Sambucus javanica* and *Plantago major*, which had the highest and second-highest for both UV and RFC values, respectively, while the most important plant families were Leguminosae and Zingiberaceae. The uses could be divided into 18 categories of musculoskeletal ailments. Muscular pain had highest prevalence among the Karen communities. 

Our review can lead to the discovery of the alternative medicines to treat MSDs. Future investigations of phytochemical compounds and pharmacological research are needed to confirm the efficacy of treatments that are part of traditional knowledge. Finally, besides medicinal information, this review emphasizes the importance of traditional knowledge.

## Figures and Tables

**Table 1:** Medicinal plants used to treat Musculoskeletal disorders (MSDs) among the Karen ethnic minority in Thailand.

Scientific Name	Family	Habit	UV	RFC	Part Used	Preparation	Administration	MSD Categories
*Acanthus montanus* (Nees) T. Anderson	ACANTHACEAE	Tree	0.037	0.037	Leaves	Decoction	Oral ingestion	Muscle pain
*Acmella oleracea* (L.) R.K. Jansen	ASTERACEAE	Herb	0.037	0.037	Roots	Alcoholic infusion	Oral ingestion	Muscle pain
*Ageratina adenophora* (Spreng.) R.M. King and H. Rob.	ASTERACEAE	Herb	0.037	0.037	Leaves	Burning	Poultices	Muscle pain
*Ageratum conyzoides* L.	ASTERACEAE	Herb	0.074	0.037	Whole plants	Decoction	Oral ingestion	Back symptom/complaint, Flank/axilla symptom/complaint
*Aglaia lawii* (Wight) C.J. Saldanha	MELIACEAE	Tree	0.037	0.037	Leaves	Decoction	Bath, oral ingestion	Muscle pain
*Alpinia galanga* (L.) Willd.	ZINGIBERACEAE	Herb	0.074	0.037	Roots	Decoction	Oral ingestion	Back symptom/complaint, Flank/axilla symptom/complaint
*Alpinia roxburghii* Sweet	ZINGIBERACEAE	Herb	0.074	0.037	Roots	Decoction	Bath, oral ingestion	Muscle pain
*Alstonia macrophylla* Wall. ex G. Don	APOCYNACEAE	Tree	0.037	0.037	Bark	Water infusion	Oral ingestion	Muscle pain
*Alstonia rostrata* C.E.C. Fisch.	APOCYNACEAE	Tree	0.074	0.037	Bark	Decoction, water infusion	Oral ingestion	Muscle pain
*Anredera cordifolia* (Ten.) Steenis	BASELLACEAE	Herb	0.074	0.037	Bulbil	Cook	Eaten as food	Back symptom/complaint, Flank/axilla symptom/complaint
*Antidesma bunius* (L.) Spreng.	EUPHORBIACEAE	Tree	0.074	0.037	Roots	Decoction	Oral ingestion	Back symptom/complaint, Flank/axilla symptom/complaint
*Asparagus filicinus* Buch.-Ham. ex D. Don	ASPARAGACEAE	Climber	0.074	0.074	Roots, whole plants	Decoction	Bath, oral ingestion	Muscle pain
*Baccaurea ramiflora* Lour.	EUPHORBIACEAE	Tree	0.074	0.037	Roots	Decoction	Oral ingestion	Back symptom/complaint, Flank/axilla symptom/complaint
*Betula alnoides* Buch.-Ham. ex D. Don	BETULACEAE	Tree	0.185	0.148	Bark, leaves	Alcoholic infusion, decoction, none	Eaten as food, oral ingestion	Flank/axilla symptom/complaint, muscle pain
*Biancaea sappan* (L.) Tod.	LEGUMINOSAE	Tree	0.370	0.148	Stems	Decoction	Oral ingestion	Back symptom/complaint, Flank/axilla symptom/complaint
*Bistorta paleacea* (Wall. ex Hook.f.) Yonek. and H. Ohashi	POLYGONACEAE	Herb	0.111	0.037	Roots	Decoction	Oral ingestion	Back symptom/complaint, Bursitis/tendinitis/synovitis NOS, Flank/axilla symptom/complaint
*Blumea balsamifera* (L.) DC.	ASTERACEAE	Shrub	0.407	0.074	Leaves, roots, whole plants	Burning, decoction, grind	Oral ingestion, poultices, steaming	Back symptom/complaint, Flank/axilla symptom/complaint, muscle pain, Sprain/strain of joint NOS
*Boehmeria glomerulifera* Miq.	URTICACEAE	Herb	0.037	0.037	Roots	Decoction	Oral ingestion	Muscle pain
*Brachypterum scandens* (Roxb.) Miq.	LEGUMINOSAE	Climber	0.037	0.037	Stems	Decoction	Oral ingestion	Muscle pain
*Buddleja asiatica* Lour.	SCROPHULARIACEAE	Shrub	0.074	0.037	Leaves	Decoction	Oral ingestion	Flank/axilla symptom/complaint, Leg/thigh symptom/complaint
*Canscora andrographioides* Griff. ex C.B. Clarke	GENTIANACEAE	Herb	0.037	0.037	Whole plants	Decoction	Oral ingestion	Muscle pain
*Cassytha filiformis* L.	LAURACEAE	Herb	0.111	0.074	Stems, whole plants	Alcoholic infusion, decoction	Oral ingestion	Muscle pain
*Celastrus paniculatus* Willd.	CELASTRACEAE	Climber	0.037	0.037	Aerial parts	Decoction	Oral ingestion	Muscle pain
*Centella asiatica* (L.) Urb.	APIACEAE	Herb	0.185	0.074	Leaves, whole plants	Decoction, none	Eaten as food, oral ingestion	Back symptom/complaint, Flank/axilla symptom/complaint, muscle pain
*Chloranthus erectus* (Buch.-Ham.) Verdc.	CHLORANTHACEAE	Shrub	0.037	0.037	Roots	Decoction	Oral ingestion	Flank/axilla symptom/complaint
*Chromolaena odorata* (L.) R.M. King and H. Rob.	ASTERACEAE	Herb	0.074	0.074	Roots, stems	Decoction	Oral ingestion	Muscle pain
*Cissus discolor* Blume	VITACEAE	Climber	0.037	0.037	Roots	Decoction	Oral ingestion	Muscle pain
*Citrus medica* L.	RUTACEAE	Tree	0.037	0.037	Leaves	Decoction	Oral ingestion	Muscle pain
*Clausena excavata* Burm.f.	RUTACEAE	Shrub	0.222	0.037	Inflorescences, leaves, whole plants	Burning, cook, none	Eaten as food, poultices, steaming	Back symptom/complaint, Flank/axilla symptom/complaint, muscle pain
*Clematis smilacifolia* Wall.	RANUNCULACEAE	Climber	0.037	0.037	Stems	Decoction	Oral ingestion	Muscle pain
*Clerodendrum disparifolium* Blume	LAMIACEAE	Shrub	0.037	0.037	Leaves	Grind	Poultices	Muscle symptom/complaint NOS
*Clerodendrum indicum* (L.) Kuntze	LAMIACEAE	Shrub	0.037	0.037	Inflorescences, leaves	Decoction	Oral ingestion	Muscle pain
*Cnestis palala* (Lour.) Merr.	CONNARACEAE	Climber	0.037	0.037	Roots	Decoction	Oral ingestion	Muscle pain
*Codariocalyx motorius* (Houtt.) H. Ohashi	LEGUMINOSAE	Shrub	0.037	0.037	Roots	Decoction	Oral ingestion	Muscle pain
*Coix lacryma-jobi* L. var. *monilifer* Watt	POACEAE	Grass	0.148	0.074	Whole plants, roots	Decoction	Oral ingestion	Back symptom/complaint, Flank/axilla symptom/complaint, muscle pain
*Crateva religiosa* G. Forst.	CAPPARACEAE	Tree	0.037	0.037	Leaves	Grind	Poultices	Sprain/strain of ankle
*Cratoxylum formosum* (Jacq.) Benth. and Hook.f. ex Dyer subsp. *pruniflorum* (Kurz) Gogelein	HYPERICACEAE	Tree	0.037	0.037	Roots, stems	Decoction	Oral ingestion	Muscle pain
*Crinum asiaticum* L.	AMARYLLIDACEAE	Herb	0.111	0.111	Leaves	Burning	Oral ingestion, poultices	Muscle pain, sprain/strain of joint NOS
*Croton kongensis* Gagnep.	EUPHORBIACEAE	Shrub	0.037	0.037	Leaves, roots	Decoction	Oral ingestion	Muscle pain
*Croton mangelong* Y.T. Chang	EUPHORBIACEAE	Shrub	0.074	0.074	Leaves	Decoction	Oral ingestion	Muscle pain
*Curcuma elata* Roxb.	ZINGIBERACEAE	Herb	0.074	0.037	Roots	Grind	Poultices	Hand/finger symptom/complaint, knee symptom/complaint
*Curcuma longa* L.	ZINGIBERACEAE	Herb	0.111	0.111	Roots	Burning, grind	Poultices	Fracture: other, leg/thigh symptom/complaint
*Curcuma zedoaria* (Christm.) Roscoe	ZINGIBERACEAE	Herb	0.074	0.037	Roots	None	Chewing	Back symptom/complaint, flank/axilla symptom/complaint
*Cuscuta chinensis* Lam.	CONVOLVULACEAE	Herb	0.037	0.037	Stems	Decoction	Oral ingestion	Muscle pain
*Cyclocodon celebicus* (Blume) D.Y. Hong	CAMPANULACEAE	Shrub	0.037	0.037	Roots	Decoction	Oral ingestion	Muscle pain
*Cymbopogon citratus* (DC.) Stapf	POACEAE	Grass	0.074	0.074	Stems, whole plants	Burning, grind	Poultices	Fracture: other, muscle pain
*Dendrocalamus brandisii* (Munro) Kurz	POACEAE	Bamboo	0.037	0.037	Roots	Decoction	Oral ingestion	Muscle pain
*Dendrophthoe pentandra* (L.) Miq.	LORANTHACEAE	Shrub	0.037	0.037	Stems	Decoction	Oral ingestion	Knee symptom/complaint
*Desmos macrocarpus* Bân	ANNONACEAE	Climber	0.037	0.037	Roots	Decoction	Oral ingestion	Muscle pain
*Dimetia ampliflora* (Hance) Neupane and N. Wikstr.	RUBIACEAE	Herb	0.074	0.037	Roots, whole plants	Decoction	Oral ingestion, steaming	Muscle pain
*Diplazium esculentum* (Retz.) Sw.	ATHYRIACEAE	Fern	0.037	0.037	Roots	Decoction	Poultices	Sprain/strain of joint NOS
*Dischidia nummularia* R. Br.	APOCYNACEAE	Herb	0.074	0.074	Leaves	Decoction, grind, none	Eaten as food, oral ingestion, poultices	Knee symptom/complaint, muscle pain
*Dracaena fragrans* (L.) Ker Gawl.	ASPARAGACEAE	Shrub	0.037	0.037	Leaves	Burning	Poultices	Sprain/strain of joint NOS
*Dracaena terniflora* Roxb.	ASPARAGACEAE	Shrub	0.037	0.037	Leaves, stems	Decoction	Oral ingestion	Muscle pain
*Duabanga grandiflora* (DC.) Walp.	LYTHRACEAE	Tree	0.074	0.074	Bark	Decoction	Oral ingestion	Muscle pain
*Dufrenoya collettii* (Gamble) Stauffer	SANTALACEAE	Herb	0.148	0.037	Roots, whole plants	Decoction	Liniment, oral ingestion, poultices	Flank/axilla symptom/complaint, muscle pain, sprain/strain of joint NOS
*Dufrenoya sessilis* (Craib) Stauffer	SANTALACEAE	Shrub	0.111	0.037	Leaves, stems	Burning, decoction	Oral ingestion, poultices	Leg/thigh symptom/complaint, muscle pain, sprain/strain of joint NOS
*Duhaldea cappa* (Buch.-Ham. ex D. Don) Pruski and Anderb.	ASTERACEAE	Shrub	0.407	0.259	Inflorescences, leaves, roots	Burning, decoction, grind	Oral ingestion, poultices	Joint symptom/complaint NOS, knee symptom/complaint, muscle pain, sprain/strain of joint NOS
*Elephantopus scaber* L.	ASTERACEAE	Herb	0.222	0.222	Roots, whole plants	Decoction	Oral ingestion	Flank/axilla symptom/complaint, muscle pain
*Eleutherine bulbosa* (Mill.) Urb.	IRIDACEAE	Herb	0.037	0.037	Roots	Grind	Liniment	Muscle pain
*Embelia ribes* Burm.f.	PRIMULACEAE	Climber	0.037	0.037	Roots	Decoction	Oral ingestion	Muscle pain
*Engelhardia spicata* Lesch. ex Blume	JUGLANDACEAE	Tree	0.074	0.074	Bark, stems	Decoction	Oral ingestion	Muscle pain
*Ensete glaucum* (Roxb.) Cheesman	MUSACEAE	Herb	0.037	0.037	Seeds	Decoction	Compress	Muscle pain
*Equisetum ramosissimum* Desf. subsp. *debile* (Roxb. ex Vaucher) Hauke	EQUISETACEAE	Low vascular plant	0.037	0.037	Stems	Decoction	Oral ingestion	Muscle pain
*Erythrina subumbrans* (Hassk.) Merr.	LEGUMINOSAE	Tree	0.074	0.074	Bark, leaves	Burning, decoction	Oral ingestion, poultices	Fracture: radius/ulna, leg/thigh symptom/complaint
*Eurycoma longifolia* Jack	SIMAROUBACEAE	Shrub	0.074	0.074	Whole plants	Decoction	Oral ingestion	Muscle pain
*Ficus semicordata* Buch.-Ham. ex Sm.	MORACEAE	Tree	0.037	0.037	Stems	Decoction	Oral ingestion	Muscle pain
*Flacourtia jangomas* (Lour.) Raeusch.	SALICACEAE	Tree	0.037	0.037	Bark	Decoction	Oral ingestion	Muscle pain
*Flacourtia rukam* Zoll. and Moritzi	SALICACEAE	Tree	0.148	0.074	Roots	Decoction	Oral ingestion	Bursitis/tendinitis/synovitis NOS, muscle pain
*Flemingia strobilifera* (L.) W.T. Aiton	LEGUMINOSAE	Shrub	0.037	0.037	Roots	Decoction	Oral ingestion	Muscle pain
*Flueggea leucopyrus* Willd.	PHYLLANTHACEAE	Shrub	0.037	0.037	Roots	Decoction	Oral ingestion	Muscle pain
*Gmelina arborea* Roxb.	LAMIACEAE	Tree	0.333	0.296	Bark, inflorescences	Burning, decoction	Oral ingestion, poultices, soak	Fracture: other, knee symptom/complaint, Muscle pain
*Gynostemma pentaphyllum* (Thunb.) Makino	CUCURBITACEAE	Climber	0.037	0.037	Whole plants	Decoction	Poultices	Muscle pain
*Heliciopsis terminalis* (Kurz) Sleumer	PROTEACEAE	Tree	0.037	0.037	Bark	Decoction	Oral ingestion	Muscle pain
*Hellenia speciosa* (J. Koenig) S.R. Dutta	COSTACEAE	Herb	0.037	0.037	Roots	Decoction	Oral ingestion	Flank/axilla symptom/complaint
*Hiptage benghalensis* (L.) Kurz	MALPIGHIACEAE	Climber	0.111	0.074	Bark, roots, stems	Decoction	Oral ingestion	Back symptom/complaint, flank/axilla symptom/complaint, muscle pain
*Hiptage benghalensis* (L.) Kurz subsp. *candicans* (Hook.f.) Sirirugsa	MALPIGHIACEAE	Shrub	0.037	0.037	Bark	Decoction	Oral ingestion	Flank/axilla symptom/complaint
*Huangtcia renifolia* (L.) H. Ohashi and K. Ohashi	LEGUMINOSAE	Shrub	0.037	0.037	Whole plants	Decoction	Oral ingestion	Muscle pain
*Hydrocotyle javanica* Thunb.	ARALIACEAE	Herb	0.111	0.074	Leaves, whole plants	Decoction, none	Eaten as food, oral ingestion	Back symptom/complaint, flank/axilla symptom/complaint, muscle pain
*Hymenasplenium apogamum* (N. Murak. and Hatan.) Nakaike	ASPLENIACEAE	Fern	0.037	0.037	Leaves	Burning	Poultices	Sprain/strain of ankle
*Illigera trifoliata* (Griff.) Dunn	HERNANDIACEAE	Climber	0.074	0.037	Leaves, whole plants	Decoction	Oral ingestion, steaming	Muscle pain
*Imperata cylindrica* (L.) Raeusch.	POACEAE	Grass	0.074	0.037	Roots	Decoction	Oral ingestion	Back symptom/complaint, flank/axilla symptom/complaint
*Indigofera caloneura* Kurz	LEGUMINOSAE	Shrub	0.037	0.037	Whole plants	Decoction	Oral ingestion	Knee symptom/complaint
*Ixora henryi* H. Lév.	RUBIACEAE	Tree	0.037	0.037	Leaves	Decoction	Oral ingestion	Muscle pain
*Kaempferia rotunda* L.	ZINGIBERACEAE	Herb	0.037	0.037	Roots	Grind	Compress	Muscle pain, knee symptom/complaint
*Leea indica* (Burm.f.) Merr.	VITACEAE	Shrub	0.074	0.074	Leaves, roots	Decoction	Oral ingestion	Knee symptom/complaint
*Lilium primulinum* Baker var. *burmanicum* (W.W. Sm.) Stearn	LILIACEAE	Herb	0.074	0.037	Roots	Decoction	Bath	Back symptom/complaint, flank/axilla symptom/complaint
*Litsea martabanica* (Kurz) Hook.f.	LAURACEAE	Tree	0.037	0.037	Whole plants	Decoction	Oral ingestion	Muscle pain
*Lycopodiella cernua* cernua (L.) Pic. Serm.	LYCOPODIACEAE	Fern	0.037	0.037	Stems	Decoction	Oral ingestion	Muscle pain
*Lygodium flexuosum* (L.) Sw.	LYGODIACEAE	Fern	0.259	0.148	Aerial parts, roots, whole plants	Decoction	Oral ingestion	Back symptom/complaint, flank/axilla symptom/complaint, leg/thigh symptom/complaint
*Macaranga denticulata* (Blume) Müll. Arg.	EUPHORBIACEAE	Tree	0.037	0.037	Roots	Decoction	Oral ingestion	Fracture: femur
*Maesa glomerata* K. Larsen and C.M. Hu	PRIMULACEAE	Shrub	0.037	0.037	Roots	Decoction	Oral ingestion	Muscle pain
*Mangifera indica* L.	ANACARDIACEAE	Tree	0.037	0.037	Stems	Decoction	Oral ingestion	Muscle pain
*Mansoa alliacea* (Lam.) A. Gentry	BIGNONIACEAE	Climber	0.037	0.037	Leaves	Decoction	Oral ingestion	Joint symptom/complaint NOS
*Melicope lunu-ankenda* (Gaertn.) T.G. Hartley	RUTACEAE	Shrub	0.111	0.037	Leaves, whole plants	Decoction, none	Bath, poultices	Back symptom/complaint, flank/axilla symptom/complaint, muscle pain
*Melicope pteleifolia* (Champ. ex Benth.) T.G. Hartley	RUTACEAE	Tree	0.037	0.037	Roots	Decoction	Oral ingestion	Muscle pain
*Memecylon pauciflorum* Blume	MELASTOMATACEAE	Shrub	0.037	0.037	Leaves	Decoction	Oral ingestion	Muscle pain
*Microcos paniculata* L.	MALVACEAE	Tree	0.037	0.037	Leaves, roots	Decoction	Oral ingestion	Muscle pain
*Miliusa thorelii* Finet and Gagnep.	ANNONACEAE	Shrub	0.704	0.259	Bark, leaves, roots, stems	Alcoholic infusion, decoction	Oral ingestion	Fracture: other, Joint symptom/complaint NOS, Knee symptom/complaint, Muscle pain
*Miliusa velutina* (Dunal) Hook.f. and Thomson	ANNONACEAE	Tree	0.074	0.037	Roots	Decoction	Oral ingestion	Back symptom/complaint, flank/axilla symptom/complaint
*Mimosa pudica* L.	LEGUMINOSAE	Herb	0.111	0.111	Roots, whole plants	Decoction	Oral ingestion, soak	Muscle pain
*Mitragyna rotundifolia* (Roxb.) Kuntze	RUBIACEAE	Tree	0.037	0.037	Roots, stems	Decoction	Oral ingestion	Rheumatoid/seropositive arthritis
*Momordica charantia* L.	CUCURBITACEAE	Climber	0.037	0.037	Whole plants	Cook	Eaten as food	Muscle pain
*Monosis volkameriifolia* (DC.) H. Rob. and Skvarla	ASTERACEAE	Shrub	0.074	0.037	Roots, stems	Decoction	Oral ingestion	Leg/thigh symptom/complaint, muscle pain
*Mussaenda sanderiana* Ridl.	RUBIACEAE	Shrub	0.037	0.037	Roots	Decoction	Poultices	Muscle pain
*Nyctocalos brunfelsiiflora* Teijsm. and Binn.	BIGNONIACEAE	Climber	0.074	0.037	Roots, stems, whole plants	Decoction	Oral ingestion	Flank/axilla symptom/complaint, muscle pain
*Oenanthe javanica* (Blume) DC.	APIACEAE	Herb	0.037	0.037	Leaves	None	Eaten as food	Muscle pain
*Oroxylum indicum* (L.) Benth. ex Kurz	BIGNONIACEAE	Tree	0.074	0.074	Bark, stems	Decoction, none	Chewing, oral ingestion	Muscle pain
*Orthosiphon aristatus* (Blume) Miq.	LAMIACEAE	Herb	0.037	0.037	Roots	Decoction	Oral ingestion	Muscle pain
*Osbeckia chinensis* L.	MELASTOMATACEAE	Shrub	0.037	0.037	Roots, whole plants	Decoction	Oral ingestion	Muscle pain
*Oxyceros bispinosus* (Griff.) Tirveng.	RUBIACEAE	Shrub	0.037	0.037	Stems	Decoction	Oral ingestion	Muscle pain
*Paris polyphylla* Sm.	MELANTHIACEAE	Herb	0.111	0.074	Roots	Alcoholic infusion, decoction	Oral ingestion	Flank/axilla symptom/complaint, muscle pain
*Peliosanthes caesia* J.M.H. Shaw	ASPARAGACEAE	Herb	0.037	0.037	Leaves, whole plants	Decoction	Oral ingestion	Neck symptom/complain
*Phlogacanthus curviflorus* Nees	ACANTHACEAE	Shrub	0.148	0.074	Inflorescences, leaves, whole plants	Burning, none	Eaten as food, poultices	Muscle pain
*Phyllanthus amarus* Schumach. and Thonn.	PHYLLANTHACEAE	Herb	0.111	0.111	Whole plants	Decoction	Oral ingestion	Muscle pain
*Phyllanthus emblica* L.	PHYLLANTHACEAE	Tree	0.037	0.037	Bark	Decoction	Oral ingestion	Muscle pain
*Phyllodium pulchellum* (L.) Desv.	LEGUMINOSAE	Shrub	0.074	0.074	Roots, whole plants	Decoction	Oral ingestion	Muscle pain
*Picrasma javanica* Blume	SIMAROUBACEAE	Tree	0.037	0.037	Bark	Water infusion	Oral ingestion	Muscle pain
*Piper boehmeriifolium* (Miq.) C. DC.	PIPERACEAE	Climber	0.037	0.037	Roots	Decoction	Oral ingestion	Muscle pain
*Piper interruptum* Opiz	PIPERACEAE	Climber	0.074	0.074	Stems	Decoction	Oral ingestion	Muscle pain
*Piper nigrum* L.	PIPERACEAE	Climber	0.074	0.037	Infructescences	Decoction	Oral ingestion	Back symptom/complaint, flank/axilla symptom/complaint
*Piper retrofractum* Vahl	PIPERACEAE	Climber	0.074	0.037	Infructescences	Decoction	Oral ingestion	Back symptom/complaint, flank/axilla symptom/complaint
*Piper ribesioides* (Wall.) C. DC	PIPERACEAE	Climber	0.037	0.037	Stems	Grind	Oral ingestion	Muscle pain
*Plantago major* L.	PLANTAGINACEAE	Herb	0.852	0.370	Leaves, roots, whole plants	Burning, grind, decoction, none, pounded	Compress, eaten as food, oral ingestion, poultices	Back symptom/complaint, flank/axilla symptom/complaint, hand/finger symptom/complaint, joint symptom/complaint NOS, knee symptom/complaint, muscle pain, sprain/strain of joint NOS
*Plumbago indica* L.	PLUMBAGINACEAE	Herb	0.148	0.074	Roots	Alcoholic infusion, decoction	Oral ingestion	Flank/axilla symptom/complaint, knee symptom/complaint, muscle pain
*Plumbago zeylanica* L.	PLUMBAGINACEAE	Shrub	0.074	0.037	Roots	Alcoholic infusion	Oral ingestion	Back symptom/complaint, flank/axilla symptom/complaint
*Plumeria obtusa* L.	APOCYNACEAE	Tree	0.037	0.037	Leaves	Decoction	Oral ingestion	Muscle pain
*Plumeria rubra* L.	APOCYNACEAE	Tree	0.074	0.037	Bark	Decoction, water infusion	Oral ingestion	Muscle pain
*Polygala arillata* Buch.-Ham. ex D. Don	POLYGALACEAE	Shrub	0.037	0.037	Inflorescences, roots	Decoction	Oral ingestion	Muscle pain
*Polygala chinensis* L.	POLYGALACEAE	Herb	0.074	0.074	Whole plants	Burning, decoction	Oral ingestion	Muscle pain
*Pothos chinensis* (Raf.) Merr.	ARACEAE	Climber	0.111	0.074	Leaves, stems, whole plants	Decoction	Oral ingestion	Leg/thigh symptom/complaint, muscle pain
*Pothos scandens* L.	ARACEAE	Climber	0.630	0.259	Whole plants	Alcoholic infusion, decoction	Oral ingestion	Back symptom/complaint, flank/axilla symptom/complaint, muscle pain, sprain/strain of joint NOS
*Psychotria yunnanensis* Hutch.	RUBIACEAE	Shrub	0.037	0.037	Stems	Decoction	Oral ingestion	Muscle pain
*Putranjiva roxburghii* Wall.	PUTRANJIVACEAE	Tree	0.037	0.037	Leaves	Burning	Poultices	Muscle pain
*Rhinacanthus nasutus* (L.) Kurz	ACANTHACEAE	Shrub	0.037	0.037	Whole plants	Decoction	Oral ingestion	Muscle pain
*Rotheca serrata* Steane and Mabb.	LAMIACEAE	Shrub	0.111	0.111	Barks, leaves	Decoction, grind	Oral ingestion, poultices	Muscle pain
*Rubia cordifolia* L.	RUBIACEAE	Herb	0.037	0.037	Whole plants	Decoction	Oral ingestion	Muscle pain
*Saccharum officinarum* L.	POACEAE	Grass	0.074	0.037	Leaves, stems	Decoction	Oral ingestion	Back symptom/complaint, flank/axilla symptom/complaint
*Salacia chinensis* L.	CELASTRACEAE	Shrub	0.037	0.037	Stems	Alcoholic infusion	Oral ingestion	Muscle pain
*Salacia verrucosa* Wight	CELASTRACEAE	Climber	0.111	0.074	Roots	Decoction	Oral ingestion	Leg/thigh symptom/complaint, muscle pain
*Sambucus javanica* Reinw. ex Blume	ADOXACEAE	Shrub	1.148	0.593	Leaves, roots, whole plants	Burning, grind, decoction	Compress, oral ingestion, poultices	Flank/axilla symptom/complaint, fracture: other, fracture: radius/ulna, joint symptom/complaint NOS, leg/thigh symptom/complaint, muscle pain, sprain/strain of joint NOS, wrist symptom/complaint
*Sambucus simpsonii* Rehder	ADOXACEAE	Shrub	0.481	0.259	Leaves, roots	Burning, decoction	Compress, oral ingestion, poultices	Fracture: other, muscle pain, sprain/strain of joint NOS
*Sarcandra glabra* (Thunb.) Nakai var. *brachystachys* (Blume) Verdc.	CHLORANTHACEAE	Shrub	0.037	0.037	Roots	Decoction	Oral ingestion	Muscle pain
*Saurauia roxburghii* Wall.	ACTINIDIACEAE	Tree	0.037	0.037	Roots	Decoction	Decoction	Muscle pain
*Schefflera leucantha* R. Vig.	ARALIACEAE	Shrub	0.259	0.148	Stems, whole plants	Burning and decoction	Bath, oral ingestion	Back symptom/complaint, flank/axilla symptom/complaint, joint symptom/complaint NOS, muscle pain
*Schima wallichii* (DC.) Korth.	THEACEAE	Tree	0.037	0.037	Leaves	Water infusion	Oral ingestion	Muscle pain
*Scleropyrum maingayi* Hook.f.	SANTALACEAE	Tree	0.148	0.037	Whole plants	Decoction	Oral ingestion	Back symptom/complaint, flank/axilla symptom/complaint, joint symptom/complaint NOS, muscle pain
*Scleropyrum pentandrum* (Dennst.) Mabb.	SANTALACEAE	Tree	0.111	0.074	Roots	Decoction	Oral ingestion	Back symptom/complaint, muscle pain
*Scoparia dulcis* L.	PLANTAGINACEAE	Herb	0.111	0.111	Whole plants	Decoction	Oral ingestion	Muscle pain
*Senna occidentalis* (L.) Link	LEGUMINOSAE	Shrub	0.037	0.037	Seeds	Burning and decoction	Oral ingestion	Muscle pain
*Sida acuta* Burm.f.	MALVACEAE	Shrub	0.148	0.074	Roots, whole plants	Decoction	Oral ingestion	Flank/axilla symptom/complaint, muscle pain, sprain/strain of joint NOS
*Sida cordifolia* L.	MALVACEAE	Shrub	0.111	0.037	Roots	Decoction	Oral ingestion	Flank/axilla symptom/complaint, muscle pain, sprain/strain of joint NOS
*Sida rhombifolia* L.	MALVACEAE	Shrub	0.037	0.037	Leaves, roots, whole plants	Decoction	Oral ingestion	Flank/axilla symptom/complaint
*Smilax corbularia* Kunth	SMILACACEAE	Climber	0.037	0.037	Roots	Decoction	Oral ingestion	Muscle pain
*Smilax glabra* Roxb.	SMILACACEAE	Climber	0.148	0.074	Roots	Decoction	Oral ingestion	Back symptom/complaint, flank/axilla symptom/complaint, muscle pain
*Smilax griffithii* A. DC.	SMILACACEAE	Climber	0.074	0.074	Whole plants	Decoction	Oral ingestion	Muscle pain
*Smilax luzonensis* C. Presl	SMILACACEAE	Climber	0.037	0.037	Roots	Decoction	Oral ingestion	Muscle pain
*Sohmaea teres* (Wall. ex Benth.) H. Ohashi and K. Ohashi	LEGUMINOSAE	Shrub	0.074	0.037	Roots	Decoction	Oral ingestion	Back symptom/complaint, flank/axilla symptom/complaint
*Solanum erianthum* D. Don	SOLANACEAE	Shrub	0.074	0.037	Stems	Decoction	Oral ingestion	Back symptom/complaint, flank/axilla symptom/complaint
*Styrax benzoides* Craib	STYRACACEAE	Tree	0.037	0.037	Roots	Decoction	Oral ingestion	Muscle pain
*Tadehagi triquetrum* (L.) H. Ohashi	LEGUMINOSAE	Shrub	0.222	0.111	Roots, whole plants	Alcoholic infusion, decoction	Oral ingestion	Back symptom/complaint, flank/axilla symptom/complaint, muscle pain
*Tetrastigma cruciatum* Craib and Gagnep.	VITACEAE	Climber	0.037	0.037	Roots	Decoction	Oral ingestion	Muscle pain
*Thunbergia coccinea* Wall. ex D. Don	ACANTHACEAE	Climber	0.148	0.074	Stems, whole plants	Decoction	Oral ingestion	Arm symptom/complaint, knee symptom/complaint, muscle pain
*Thunbergia laurifolia* Lindl.	ACANTHACEAE	Climber	0.111	0.111	Leaves, roots, stems, whole plants	Decoction	Oral ingestion	Muscle pain
*Thysanolaena latifolia* (Roxb. ex Hornem.) Honda	POACEAE	Grass	0.037	0.037	Roots	Decoction	Oral ingestion	Muscle pain
*Tinospora crispa* (L.) Hook.f. and Thomson	MENISPERMACEAE	Climber	0.185	0.148	Aerial parts, stems	Alcoholic infusion, sugar infusion, decoction	Oral ingestion, poultices	Back symptom/complaint, flank/axilla symptom/complaint, muscle pain
*Tupistra muricata* (Gagnep.) N. Tanaka	ASPARAGACEAE	Herb	0.222	0.074	Leaves, roots	Burning, decoction	Poultices	Fracture: femur, leg/thigh symptom/complaint, sprain/strain of joint NOS, wrist symptom/complaint
*Turpinia pomifera* (Roxb.) DC.	STAPHYLEACEAE	Tree	0.074	0.037	Roots	Alcoholic infusion, decoction	Oral ingestion	Muscle pain
*Uncaria laevigata* Wall. ex G. Don	RUBIACEAE	Climber	0.037	0.037	Roots	Decoction	Oral ingestion	Muscle pain
*Xantolis burmanica* (Collett and Hemsl.) P. Royen	SAPOTACEAE	Tree	0.037	0.037	Bark	Decoction	Oral ingestion	Muscle pain
*Zingiber latifolium* Theilade and Mood	ZINGIBERACEAE	Shrub	0.037	0.037	Roots	Decoction	Oral ingestion	Fracture: other
*Zingiber officinale* Roscoe	ZINGIBERACEAE	Shrub	0.037	0.037	Roots	Grind	Oral ingestion	Muscle pain
*Zingiber ottensii* Valeton	ZINGIBERACEAE	Shrub	0.111	0.074	Roots	Decoction	Oral ingestion	Back symptom/complaint, flank/axilla symptom/complaint, muscle pain
*Zingiber purpureum* Roscoe	ZINGIBERACEAE	Shrub	0.074	0.074	Roots	Concoction, grind	Poultices	Muscle pain, sprain/strain of joint NOS
*Ziziphus cambodianus* Pierre	RHAMNACEAE	Tree	0.074	0.074	Barks	Decoction	Oral ingestion	Muscle pain

**Table 2:** Values for Informant Consensus Factor (ICF) recorded among Karen communities in Thailand, divided per use category following the International Classification of Primary Care [32].

Code	Category	Number of Use Reports (Nur)	Number of Species (Nt)	ICF
L12	Hand/finger symptom/complaint	5	2	0.75
L76	Fracture: other	19	7	0.67
L79	Sprain/strain of joint	32	14	0.58
L20	Joint symptom/complaint	10	5	0.56
L87	Bursitis/tendinitis/synovitis	3	2	0.50
L11	Wrist symptom/complaint	3	2	0.50
L15	Knee symptom/complaint	18	11	0.41
L18	Muscle pain	187	117	0.38
L05	Flank/axilla symptom/complaint	65	47	0.28
L02	Back symptom/complaint	44	32	0.28
L14	Leg/thigh symptom/complaint	11	10	0.10
L09	Arm symptom/complaint	1	1	0.00
L75	Fracture: femur	2	2	0.00
L72	Fracture: radius/ulna	2	2	0.00
L19	Muscle symptom/complaint	1	1	0.00
L01	Neck symptom/complain	1	1	0.00
L88	Rheumatoid/seropositive arthritis	1	1	0.00
L77	Sprain/strain of ankle	2	2	0.00

**Table 3:** The 15 references from which we extracted original data on medicinal plants species used to treat musculoskeletal system disorders among Karen communities in Thailand.

Source	Village	Subdistrict	District	Province	#Species
Junsongduang et al. [90]	Mae Hae Tai	Pang Hin Fon	Mae Chaem	Chiang Mai	8
Kaewsangsai [29]	Khun Khun Noi	Mae Tuen	Omkoi	Chiang Mai	52
Kamwong [91]	Mai Sa Wan	Ban Luang	Chom Thong	Chiang Mai	8
	Huay Poo Ling	Ban Luang	Chom Thong	Chiang Mai	15
Mahawongsanan [92]	Huai Sompoi	Doi Kaew	Chom Thong	Chiang Mai	3
Pongamornkul [93]	Yang Tung Pong	Mae Na	Chiang Dao	Chiang Mai	5
	Yang Poo To	Chiang Dao	Chiang Dao	Chiang Mai	6
Puling [94]	Mae Klang Luang	Ban Luang	Chom Thong	Chiang Mai	3
	Ang Ka Noi	Ban Luang	Chom Thong	Chiang Mai	3
Sukkho [95]	Kio Pong	Chaem Luang	Mae Chaem	Chiang Mai	13
	Chaem Noi	Ban Chan	Mae Chaem	Chiang Mai	10
	San Muang	Ban Chan	Mae Chaem	Chiang Mai	10
	Huay Bong	Ban Chan	Mae Chaem	Chiang Mai	11
	Huay Hom	Ban Chan	Mae Chaem	Chiang Mai	9
Tangjitman [96]	Huay Hea	Samoeng Tai	Samoeng District	Chiang Mai	12
	Mai Lan Kam	Samoeng Tai	Samoeng District	Chiang Mai	17
Winjchiyanan [97]	Thung Luang	Mae Wang	Mae Wang	Chiang Mai	13
	Pa Tak	Sop Poeng	Mae Tang	Chiang Mai	2
	Mae Lod Tai	Sop Poeng	Mae Tang	Chiang Mai	11
	Mae Hae Nuea	Na Chor	Mae Chaem	Chiang Mai	4
	Huay Tong	Mae Wang	Mae Wang	Chiang Mai	3
Sonsupub [98]	Rai Pa	Huay Khayen	Thongphaphume	Kanchanaburi	3
Moonjai [99]	Huay Hom	Huay Hom	Mae La Noi	Mae Hong Son	1
Trisonthi and Trisonthi [100]	Six small sub-villages (Hua Mae Surin, Hua Hua, Mae Surin Noi, Payoi, Kano, and Mae U Kor Noi)	Mae Ukho	Khun Yuam	Mae Hong Son	5
Junkhonkaen [101]	Bo Wee	Tanao Si	Suan Phueng	Ratchaburi	10
Tangjitman [102]	Huay Nam Nak	Tanao Si	Suan Phueng	Ratchaburi	3
Kantasrila [103]	Wa Do Kro	Mae Song	Tha Song Yang District	Tak	61

## Supplementary Materials

The following are available online at https://www.mdpi.com/2223-7747/9/7/811/s1, Table S1: The reference and number of pseudo informants of medicinal plants used to treat Musculoskeletal disorders (MSDs) among the Karen ethnic minority in Thailand.
