# Supplementary material for: Medicinal Plants for Treating Musculoskeletal Disorders among Karen in Thailand
Source: Plants (Basel). 2020 Jun 28;9(7):811. doi: 10.3390/plants9070811 (PMC7412036; doi:10.3390/plants9070811)
Supplement: Supplementary file 1 [file plants-09-00811-s001.pdf]

Supplementary Data

Table S1 The reference and number of pseudo informants of medicinal plants used to treat Musculoskeletal disorders (MSDs) among the Karen ethnic minority in Thailand.

**Scientific Name**	**Family**	**No. Pseudo informants**	**Part of Use**	**Preparation**	**Application**	**ICPC-2 2nd Level**	**Reference**
Acanthus montanus (Nees) T. Anderson	ACANTHACEAE	1	Leaves	Decoction	Oral ingestion	Muscle pain	[1]
Acmella oleracea (L.) R.K. Jansen	ASTERACEAE	1	Roots	Alcoholic infusion	Oral ingestion	Muscle pain	[1]
Ageratina adenophora (Spreng.) R.M. King and H. Rob.	ASTERACEAE	1	Leaves	Burning	Poultices	Muscle pain	[2]
Ageratum conyzoides L.	ASTERACEAE	1	Whole plants	Decoction	Oral ingestion	Back symptom/complaint, Flank/axilla symptom/complaint	[3]
Aglaia lawii (Wight) C.J. Saldanha	MELIACEAE	1	Leaves	Decoction	Bath, oral ingestion	Muscle pain	[4]
Alpinia galanga (L.) Willd.	ZINGIBERACEAE	1	Roots	Decoction	Oral ingestion	Back symptom/complaint, Flank/axilla symptom/complaint	[5]
Alpinia roxburghii Sweet	ZINGIBERACEAE	1	Roots	Decoction	Bath, oral ingestion	Muscle pain	[2]

Alstonia macrophylla Wall. ex G. Don	APOCYNACEAE	1	Bark	Water infusion	Oral ingestion	Muscle pain	[6]
Alstonia rostrata C.E.C. Fisch.	APOCYNACEAE	1	Bark	Decoction, water infusion	Oral ingestion	Muscle pain	[2]
Anredera cordifolia (Ten.) Steenis	BASELLACEAE	1	Bulbil	Cook	Eaten as food	Back symptom/complaint, Flank/axilla symptom/complaint	[3]
Antidesma bunius (L.) Spreng.	EUPHORBIACEAE	1	Roots	Decoction	Oral ingestion	Back symptom/complaint, Flank/axilla symptom/complaint	[5]
Asparagus filicinus Buch.-Ham. ex D. Don	ASPARAGACEAE	2	Roots, whole plants	Decoction	Bath, oral ingestion	Muscle pain	[1,5]
Baccaurea ramiflora Lour.	EUPHORBIACEAE	1	Roots	Decoction	Oral ingestion	Back symptom/complaint, Flank/axilla symptom/complaint	[5]
Betula alnoides Buch.-Ham. ex D. Don	BETULACEAE	4	Bark, leaves	Alcoholic infusion, decoction, none	Eaten as food, oral ingestion	Flank/axilla symptom/complaint, Muscle pain	[2,6,7]
Biancaea sappan (L.) Tod.	LEGUMINOSAE	1	Stems	Decoction	Oral ingestion	Back symptom/complaint, Flank/axilla symptom/complaint	[3]
Bistorta paleacea (Wall. ex	POLYGONACEAE	2	Roots	Decoction	Oral ingestion	Back symptom/compla	[3]

Hook.f.) Yonek. and H. Ohashi						int, Bursitis/tendinitis /synovitis NOS, Flank/axilla symptom/complaint	
Blumea balsamifera (L.) DC.	ASTERACEAE	1	Leaves, roots, whole plants	Burning, decoction, grind	Oral ingestion, poultices, steaming	Back symptom/complaint, Flank/axilla symptom/complaint, Muscle pain, Sprain/strain of joint NOS	[2,6]
Boehmeria glomerulifera Miq.	URTICACEAE	1	Roots	Decoction	Oral ingestion	Muscle pain	[4]
Brachypterum scandens (Roxb.) Miq.	LEGUMINOSAE	1	Stems	Decoction	Oral ingestion	Muscle pain	[8]
Buddleja asiatica Lour.	SCROPHULARIACEAE	4	Leaves	Decoction	Oral ingestion	Flank/axilla symptom/complaint, Leg/thigh symptom/complaint	[6]
Canscora andrographioides Griff. ex C.B. Clarke	GENTIANACEAE	1	Whole plants	Decoction	Oral ingestion	Muscle pain	[2]
Cassytha filiformis L.	LAURACEAE	2	Stems, whole plants	Alcoholic infusion, decoction	Oral ingestion	Muscle pain	[2,3]
Celastrus paniculatus Willd.	CELASTRACEAE	1	Aerial parts	Decoction	Oral ingestion	Muscle pain	[7]

Centella asiatica (L.) Urb.	APIACEAE	2	Leaves, whole plants	Decoction, none	Eaten as food, oral ingestion	Back symptom/complaint, Flank/axilla symptom/complaint, Muscle pain	[3,5]
Chloranthus erectus (Buch.-Ham.) Verdc.	CHLORANTHACEAE	1	Roots	Decoction	Oral ingestion	Flank/axilla symptom/complaint	[9]
Chromolaena odorata (L.) R.M. King and H. Rob.	ASTERACEAE	2	Roots, stems	Decoction	Oral ingestion	Muscle pain	[2,6]
Cissus discolor Blume	VITACEAE	1	Roots	Decoction	Oral ingestion	Muscle pain	[6]
Citrus medica L.	RUTACEAE	1	Leaves	Decoction	Oral ingestion	Muscle pain	[2]
Clausena excavata Burm.f.	RUTACEAE	1	Inflorescences, leaves, whole plants	Burning, cook, none	Eaten as food, poultices, steaming	Back symptom/complaint, Flank/axilla symptom/complaint, Muscle pain	[6]
Clematis smilacifolia Wall.	RANUNCULACEAE	1	Stems	Decoction	Oral ingestion	Muscle pain	[2]
Clerodendrum disparifolium Blume	LAMIACEAE	1	Leaves	Grind	Poultices	Muscle symptom/complaint NOS	[4]
Clerodendrum indicum (L.) Kuntze	LAMIACEAE	1	Inflorescences, leaves	Decoction	Oral ingestion	Muscle pain	[10]
Cnestis palala (Lour.) Merr.	CONNARACEAE	1	Roots	Decoction	Oral ingestion	Muscle pain	[6]
Codariocalyx motorius (Houtt.) H. Ohashi	LEGUMINOSAE	1	Roots	Decoction	Oral ingestion	Muscle pain	[6]

Coix lacryma-jobi L. var. *monilifer* Watt	POACEAE	2	Whole plants, roots	Decoction	Oral ingestion	Back symptom/complaint, Flank/axilla symptom/complaint, Muscle pain	[3]
Crateva religiosa G. Forst.	CAPPARACEAE	1	Leaves	Grind	Poultices	Sprain/strain of ankle	[6]
Cratoxylum formosum (Jacq.) Benth. and Hook.f. ex Dyer subsp. *pruniflorum* (Kurz) Gogelein	HYPERICACEAE	1	Roots, stems	Decoction	Oral ingestion	Muscle pain	[1]
Crinum asiaticum L.	AMARYLLIDACEAE	3	Leaves	Burning	Oral ingestion, poultices	Muscle pain, Sprain/strain of joint NOS	[1,8,11]
Croton kongensis Gagnep.	EUPHORBIACEAE	2	Leaves, roots	Decoction	Oral ingestion	Muscle pain	[9]
Croton mangelong Y.T. Chang	EUPHORBIACEAE	1	Leaves	Decoction	Oral ingestion	Muscle pain	[1]
Curcuma elata Roxb.	ZINGIBERACEAE	3	Roots	Grind	Poultices	Hand/finger symptom/complaint, Knee symptom/complaint	[2,5]
Curcuma longa L.	ZINGIBERACEAE	1	Roots	Burning, grind	Poultices	Fracture: other, Leg/thigh symptom/complaint	[11]
Curcuma zedoaria	ZINGIBERACEAE	1	Roots	None	Chewing	Back symptom/complaint, Flank/axilla	[6]

(Christm.) Roscoe						symptom/complaint	
Cuscuta chinensis Lam.	CONVOLVULACEAE	1	Stems	Decoction	Oral ingestion	Muscle pain	[2]
Cyclocodon celebicus (Blume) D.Y. Hong	CAMPANULACEAE	1	Roots	Decoction	Oral ingestion	Muscle pain	[6]
Cymbopogon citratus (DC.) Stapf	POACEAE	2	Stems, whole plants	Burning, grind	Poultices	Fracture: other, Muscle pain	[2,5]
Dendrocalamus brandisii (Munro) Kurz	POACEAE	1	Roots	Decoction	Oral ingestion	Muscle pain	[5]
Dendrophthoe pentandra (L.) Miq.	LORANTHACEAE	1	Stems	Decoction	Oral ingestion	Knee symptom/complaint	[1]
Desmos macrocarpus Bân	ANNONACEAE	1	Roots	Decoction	Oral ingestion	Muscle pain	[2]
Dimetia ampliflora (Hance) Neupane and N. Wikstr.	RUBIACEAE	1	Roots, whole plants	Decoction	Oral ingestion, steaming	Muscle pain	[6]
Diplazium esculentum (Retz.) Sw.	ATHYRIACEAE	1	Roots	Decoction	Poultices	Sprain/strain of joint NOS	[2]
Dischidia nummularia R. Br.	APOCYNACEAE	2	Leaves	Decoction, grind, none	Eaten as food, oral ingestion, poultices	Knee symptom/complaint, Muscle pain	[3,7]
Dracaena fragrans (L.) Ker Gawl.	ASPARAGACEAE	1	Leaves	Burning	Poultices	Sprain/strain of joint NOS	[1]

Dracaena terniflora Roxb.	ASPARAGACEAE	1	Leaves, stems	Decoction	Oral ingestion	Muscle pain	[2]
Duabanga grandiflora (DC.) Walp.	LYTHRACEAE	2	Bark	Decoction	Oral ingestion	Muscle pain	[2,5]
Dufrenoya collettii (Gamble) Stauffer	SANTALACEAE	1	Roots, whole plants	Decoction	Liniment, oral ingestion, poultices	Flank/axilla symptom/complaint, Muscle pain, Sprain/strain of joint NOS	[6]
Dufrenoya sessilis (Craib) Stauffer	SANTALACEAE	1	Leaves, stems	Burning, decoction	Oral ingestion, poultices	Leg/thigh symptom/complaint, Muscle pain, Sprain/strain of joint NOS	[2]
Duhaldea cappa (Buch.-Ham. ex D. Don) Pruski and Anderb.	ASTERACEAE	7	Inflorescences, leaves, roots	Burning, decoction, grind	Oral ingestion, poultices	Joint symptom/complaint NOS, Knee symptom/complaint, Muscle pain, Sprain/strain of joint NOS	[3,6,7]
Elephantopus scaber L.	ASTERACEAE	6	Roots, whole plants	Decoction	Oral ingestion	Flank/axilla symptom/complaint, Muscle pain	[1,2,3,5,6]
Eleutherine bulbosa (Mill.) Urb.	IRIDACEAE	1	Roots	Grind	Liniment	Muscle pain	[5]
Embelia ribes Burm.f.	PRIMULACEAE	1	Roots	Decoction	Oral ingestion	Muscle pain	[6]
Engelhardia spicata Lesch. ex Blume	JUGLANDACEAE	2	Bark, stems	Decoction	Oral ingestion	Muscle pain	[1,7]
Ensete glaucum (Roxb.) Cheesman	MUSACEAE	1	Seeds	Decoction	Compress	Muscle pain	[1]

Equisetum ramosissimum Desf. subsp. *debile* (Roxb. ex Vaucher) Hauke	EQUISETACEAE	1	Stems	Decoction	Oral ingestion	Muscle pain	[7]
Erythrina subumbrans (Hassk.) Merr.	LEGUMINOSAE	2	Bark, leaves	Burning, decoction	Oral ingestion, poultices	Fracture: radius/ulna, Leg/thigh symptom/complaint	[2,9]
Eurycoma longifolia Jack	SIMAROUBACEAE	2	Whole plants	Decoction	Oral ingestion	Muscle pain	[7]
Ficus semicordata Buch.-Ham. ex Sm.	MORACEAE	1	Stems	Decoction	Oral ingestion	Muscle pain	[8]
Flacourtia jangomas (Lour.) Raeusch.	SALICACEAE	1	Bark	Decoction	Oral ingestion	Muscle pain	[1]
Flacourtia rukam Zoll. and Moritzi	SALICACEAE	2	Roots	Decoction	Oral ingestion	Bursitis/tendinitis/synovitis NOS, Muscle pain	[5]
Flemingia strobilifera (L.) W.T. Aiton	LEGUMINOSAE	1	Roots	Decoction	Oral ingestion	Muscle pain	[6]
Flueggea leucopyrus Willd.	PHYLLANTHACEAE	1	Roots	Decoction	Oral ingestion	Muscle pain	[6]
Gmelina arborea Roxb.	LAMIACEAE	8	Bark, inflorescences	Burning, decoction	Oral ingestion, poultices, soak	Fracture: other, Knee symptom/complaint, Muscle pain	[3,4,5,6,7]
Gynostemma pentaphyllum	CUCURBITACEAE	1	Whole plants	Decoction	Poultices	Muscle pain	[6]

(Thunb.) Makino							
Heliciopsis terminalis (Kurz) Sleumer	PROTEACEAE	1	Bark	Decoction	Oral ingestion	Muscle pain	[7]
Hellenia speciosa (J. Koenig) S.R. Dutta	COSTACEAE	1	Roots	Decoction	Oral ingestion	Flank/axilla symptom/complaint	[4]
Hiptage benghalensis (L.) Kurz	MALPIGHIACEAE	1	Bark, roots, stems	Decoction	Oral ingestion	Back symptom/complaint, Flank/axilla symptom/complaint, Muscle pain	[5]
Hiptage benghalensis (L.) Kurz subsp. *candicans* (Hook.f.) Sirirugsa	MALPIGHIACEAE	2	Bark	Decoction	Oral ingestion	Flank/axilla symptom/complaint	[1,5]
Huangtcia renifolia (L.) H. Ohashi and K. Ohashi	LEGUMINOSAE	1	Whole plants	Decoction	Oral ingestion	Muscle pain	[1]
Hydrocotyle javanica Thunb.	ARALIACEAE	2	Leaves, whole plants	Decoction, none	Eaten as food, oral ingestion	Back symptom/complaint, Flank/axilla symptom/complaint, Muscle pain	[2,6]
Hymenasplenium apogamum (N. Murak. and Hatan.) Nakaike	ASPLENIACEAE	1	Leaves	Burning	Poultices	Sprain/strain of ankle	[2]
Illigera trifoliata (Griff.) Dunn	HERNANDIACEAE	1	Leaves, whole plants	Decoction	Oral ingestion, steaming	Muscle pain	[6]

Imperata cylindrica (L.) Raeusch.	POACEAE	1	Roots	Decoction	Oral ingestion	Back symptom/complaint, Flank/axilla symptom/complaint	[6]
Indigofera caloneura Kurz	LEGUMINOSAE	1	Whole plants	Decoction	Oral ingestion	Knee symptom/complaint	[3]
Ixora henryi H. Lév.	RUBIACEAE	1	Leaves	Decoction	Oral ingestion	Muscle pain	[2]
Kaempferia rotunda L.	ZINGIBERACEAE	1	Roots	Grind	Compress	Muscle pain, Knee symptom/complaint	[1]
Leea indica (Burm.f.) Merr.	VITACEAE	2	Leaves, roots	Decoction	Oral ingestion	Knee symptom/complaint	[5,12]
Lilium primulinum Baker var. *burmanicum* (W.W. Sm.) Stearn	LILIACEAE	1	Roots	Decoction	Bath	Back symptom/complaint, Flank/axilla symptom/complaint	[3]
Litsea martabanica (Kurz) Hook.f.	LAURACEAE	1	Whole plants	Decoction	Oral ingestion	Muscle pain	[6]
Lycopodiella cernua cernua (L.) Pic. Serm.	LYCOPODIACEAE	1	Stems	Decoction	Oral ingestion	Muscle pain	[5]
Lygodium flexuosum (L.) Sw.	LYGODIACEAE	4	Aerial parts, roots, whole plants	Decoction	Oral ingestion	Back symptom/complaint, Flank/axilla symptom/complaint, Leg/thigh symptom/complaint	[6,13,14]

Macaranga denticulata (Blume) Müll. Arg.	EUPHORBIACEAE	1	Roots	Decoction	Oral ingestion	Fracture: femur	[2]
Maesa glomerata K. Larsen and C.M. Hu	PRIMULACEAE	1	Roots	Decoction	Oral ingestion	Muscle pain	[6]
Mangifera indica L.	ANACARDIACEAE	1	Stems	Decoction	Oral ingestion	Muscle pain	[2]
Mansoa alliacea (Lam.) A. Gentry	BIGNONIACEAE	1	Leaves	Decoction	Oral ingestion	Joint symptom/complaint NOS	[10]
Melicope lunu-ankenda (Gaertn.) T.G. Hartley	RUTACEAE	1	Leaves, whole plants	Decoction, none	Bath, poultices	Back symptom/complaint, Flank/axilla symptom/complaint, Muscle pain	[7]
Melicope pteleifolia (Champ. ex Benth.) T.G. Hartley	RUTACEAE	1	Roots	Decoction	Oral ingestion	Muscle pain	[6]
Memecylon pauciflorum Blume	MELASTOMATACEAE	1	Leaves	Decoction	Oral ingestion	Muscle pain	[2]
Microcos paniculata L.	MALVACEAE	1	Leaves, roots	Decoction	Oral ingestion	Muscle pain	[6]
Miliusa thorelii Finet and Gagnep.	ANNONACEAE	7	Bark, leaves, roots, stems	Alcoholic infusion, decoction	Oral ingestion	Fracture: other, Joint symptom/complaint NOS, Knee symptom/complaint, Muscle pain	[3,7]
Miliusa velutina (Dunal) Hook.f. and Thomson	ANNONACEAE	1	Roots	Decoction	Oral ingestion	Back symptom/complaint, Flank/axilla	[9]

						symptom/complaint	
Mimosa pudica L.	LEGUMINOSAE	3	Roots, whole plants	Decoction	Oral ingestion, soak	Muscle pain	[2,6,7]
Mitragyna rotundifolia (Roxb.) Kuntze	RUBIACEAE	1	Roots, stems	Decoction	Oral ingestion	Rheumatoid/seropositive arthritis	[4]
Momordica charantia L.	CUCURBITACEAE	1	Whole plants	Cook	Eaten as food	Muscle pain	[6]
Monosis volkameriifolia (DC.) H. Rob. and Skvarla	ASTERACEAE	1	Roots, stems	Decoction	Oral ingestion	Leg/thigh symptom/complaint, Muscle pain	[5]
Mussaenda sanderiana Ridl.	RUBIACEAE	1	Roots	Decoction	Poultices	Muscle pain	[7]
Nyctocalos brunfelsiiflora Teijsm. and Binn.	BIGNONIACEAE	1	Roots, stems, whole plants	Decoction	Oral ingestion	Flank/axilla symptom/complaint, Muscle pain	[6]
Oenanthe javanica (Blume) DC.	APIACEAE	1	Leaves	None	Eaten as food	Muscle pain	[2]
Oroxylum indicum (L.) Benth. ex Kurz	BIGNONIACEAE	2	Bark, stems	Decoction, none	Chewing, oral ingestion	Muscle pain	[2,6]
Orthosiphon aristatus (Blume) Miq.	LAMIACEAE	1	Roots	Decoction	Oral ingestion	Muscle pain	[8]
Osbeckia chinensis L.	MELASTOMATACEAE	1	Roots, whole plants	Decoction	Oral ingestion	Muscle pain	[6]
Oxyceros bispinosus (Griff.) Tirveng.	RUBIACEAE	1	Stems	Decoction	Oral ingestion	Muscle pain	[8]

Paris polyphylla Sm.	MELANTHIACEAE	2	Roots	Alcoholic infusion, decoction	Oral ingestion	Flank/axilla symptom/complaint, Muscle pain	[3]
Peliosanthes caesia J.M.H. Shaw	ASPARAGACEAE	1	Leaves, whole plants	Decoction	Oral ingestion	Neck symptom/complain	[6]
Phlogacanthus curviflorus Nees	ACANTHACEAE	2	Inflorescences, leaves, whole plants	Burning, none	Eaten as food, poultices	Muscle pain	[6,9]
Phyllanthus amarus Schumach. and Thonn.	PHYLLANTHACEAE	1	Whole plants	Decoction	Oral ingestion	Muscle pain	[2]
Phyllanthus emblica L.	PHYLLANTHACEAE	3	Bark	Decoction	Oral ingestion	Muscle pain	[9,8]
Phyllodium pulchellum (L.) Desv.	LEGUMINOSAE	2	Roots, whole plants	Decoction	Oral ingestion	Muscle pain	[1,7]
Picrasma javanica Blume	SIMAROUBACEAE	1	Bark	Water infusion	Oral ingestion	Muscle pain	[6]
Piper boehmeriifolium (Miq.) C. DC.	PIPERACEAE	1	Roots	Decoction	Oral ingestion	Muscle pain	[2]
Piper interruptum Opiz	PIPERACEAE	2	Stems	Decoction	Oral ingestion	Muscle pain	[1]
Piper nigrum L.	PIPERACEAE	1	Infructescences	Decoction	Oral ingestion	Back symptom/complaint, Flank/axilla symptom/complaint	[5]
Piper retrofractum Vahl	PIPERACEAE	1	Infructescences	Decoction	Oral ingestion	Back symptom/complaint, Flank/axilla symptom/complaint	[5]

Piper ribesioides (Wall.) C. DC	PIPERACEAE	1	Stems	Grind	Oral ingestion	Muscle pain	[5]
Plantago major L.	PLANTAGINACEAE	10	Leaves, roots, whole plants	Burning, grind, decoction, none, pounded	Compress, eaten as food, oral ingestion, poultices	Back symptom/complaint, Flank/axilla symptom/complaint, Hand/finger symptom/complaint, Joint symptom/complaint NOS, Knee symptom/complaint, Muscle pain, Sprain/strain of joint NOS	[3,5,7,9,13,14]
Plumbago indica L.	PLUMBAGINACEAE	2	Roots	Alcoholic infusion, decoction	Oral ingestion	Flank/axilla symptom/complaint, Knee symptom/complaint, Muscle pain	[3,9]
Plumbago zeylanica L.	PLUMBAGINACEAE	1	Roots	Alcoholic infusion	Oral ingestion	Back symptom/complaint, Flank/axilla symptom/complaint	[3]
Plumeria obtusa L.	APOCYNACEAE	1	Leaves	Decoction	Oral ingestion	Muscle pain	[2]
Plumeria rubra L.	APOCYNACEAE	1	Bark	Decoction, water infusion	Oral ingestion	Muscle pain	[6]
Polygala arillata Buch.-Ham. ex D. Don	POLYGALACEAE	2	Inflorescences, roots	Decoction	Oral ingestion	Muscle pain	[2,12]
Polygala chinensis L.	POLYGALACEAE	1	Whole plants	Burning, decoction	Oral ingestion	Muscle pain	[6]

Pothos chinensis (Raf.) Merr.	ARACEAE	2	Leaves, stems, whole plants	Decoction	Oral ingestion	Leg/thigh symptom/complaint, Muscle pain	[2,6]
Pothos scandens L.	ARACEAE	7	Whole plants	Alcoholic infusion, decoction	Oral ingestion	Back symptom/complaint, Flank/axilla symptom/complaint, Muscle pain, Sprain/strain of joint NOS	[3,7]
Psychotria yunnanensis Hutch.	RUBIACEAE	1	Stems	Decoction	Oral ingestion	Muscle pain	[2]
Putranjiva roxburghii Wall.	PUTRANJIVACEAE	1	Leaves	Burning	Poultices	Muscle pain	[8]
Rhinacanthus nasutus (L.) Kurz	ACANTHACEAE	1	Whole plants	Decoction	Oral ingestion	Muscle pain	[5]
Rotheca serrata Steane and Mabb.	LAMIACEAE	3	Barks, leaves	Decoction, grind	Oral ingestion, poultices	Muscle pain	[4,6,7]
Rubia cordifolia L.	RUBIACEAE	1	Whole plants	Decoction	Oral ingestion	Muscle pain	[6]
Saccharum officinarum L.	POACEAE	1	Leaves, stems	Decoction	Oral ingestion	Back symptom/complaint, Flank/axilla symptom/complaint	[5]
Salacia chinensis L.	CELASTRACEAE	1	Stems	Alcoholic infusion	Oral ingestion	Muscle pain	[8]
Salacia verrucosa Wight	CELASTRACEAE	2	Roots	Decoction	Oral ingestion	Leg/thigh symptom/complaint, Muscle pain	[2,6]
Sambucus javanica Reinw. ex Blume	ADOXACEAE	16	Leaves, roots, whole plants	Burning, grind, decoction	Compress, oral	Flank/axilla symptom/complaint, Fracture:	[2,3,4,6,7,9,12,13,14,15]

					ingestion, poultices	other, Fracture: radius/ulna, Joint symptom/complaint NOS, Leg/thigh symptom/complaint, Muscle pain, Sprain/strain of joint NOS, Wrist symptom/complaint	
Sambucus simpsonii Rehder	ADOXACEAE	7	Leaves, roots	Burning, decoction	Compress, oral ingestion, poultices	Fracture: other, Muscle pain, Sprain/strain of joint NOS	[1,3]
Sarcandra glabra (Thunb.) Nakai var. *brachystachys* (Blume) Verdc.	CHLORANTHACEAE	1	Roots	Decoction	Oral ingestion	Muscle pain	[6]
Saurauia roxburghii Wall.	ACTINIDIACEAE	1	Roots	Decoction	Decoction	Muscle pain	[2]
Schefflera leucantha R. Vig.	ARALIACEAE	4	Stems, whole plants	Burning and decoction	Bath, oral ingestion	Back symptom/complaint, Flank/axilla symptom/complaint, Joint symptom/complaint NOS, Muscle pain	[3,8]
Schima wallichii (DC.) Korth.	THEACEAE	1	Leaves	Water infusion	Oral ingestion	Muscle pain	[5]
Scleropyrum maingayi Hook.f.	SANTALACEAE	1	Whole plants	Decoction	Oral ingestion	Back symptom/complaint, Flank/axilla symptom/compla	[6]

						int, Joint symptom/complaint NOS, Muscle pain	
Scleropyrum pentandrum (Dennst.) Mabb.	SANTALACEAE	2	Roots	Decoction	Oral ingestion	Back symptom/complaint, Muscle pain	[2,6]
Scoparia dulcis L.	PLANTAGINACEAE	3	Whole plants	Decoction	Oral ingestion	Muscle pain	[1,2,6]
Senna occidentalis (L.) Link	LEGUMINOSAE	1	Seeds	Burning and decoction	Oral ingestion	Muscle pain	[1]
Sida acuta Burm.f.	MALVACEAE	2	Roots, whole plants	Decoction	Oral ingestion	Flank/axilla symptom/complaint, Muscle pain, Sprain/strain of joint NOS	[2,6]
Sida cordifolia L.	MALVACEAE	1	Roots	Decoction	Oral ingestion	Flank/axilla symptom/complaint, Muscle pain, Sprain/strain of joint NOS	[6]
Sida rhombifolia L.	MALVACEAE	1	Leaves, roots, whole plants	Decoction	Oral ingestion	Flank/axilla symptom/complaint	[6]
Smilax corbularia Kunth	SMILACACEAE	1	Roots	Decoction	Oral ingestion	Muscle pain	[2]
Smilax glabra Roxb.	SMILACACEAE	2	Roots	Decoction	Oral ingestion	Back symptom/complaint, Flank/axilla symptom/complaint, Muscle pain	[2,6]
Smilax griffithii A. DC.	SMILACACEAE	2	Whole plants	Decoction	Oral ingestion	Muscle pain	[7]

Smilax luzonensis C. Presl	SMILACACEAE	1	Roots	Decoction	Oral ingestion	Muscle pain	[2]
Sohmaea teres (Wall. ex Benth.) H. Ohashi and K. Ohashi	LEGUMINOSAE	1	Roots	Decoction	Oral ingestion	Back symptom/complaint, Flank/axilla symptom/complaint	[12]
Solanum erianthum D. Don	SOLANACEAE	1	Stems	Decoction	Oral ingestion	Back symptom/complaint, Flank/axilla symptom/complaint	[3]
Styrax benzoides Craib	STYRACACEAE	1	Roots	Decoction	Oral ingestion	Muscle pain	[2]
Tadehagi triquetrum (L.) H. Ohashi	LEGUMINOSAE	3	Roots, whole plants	Alcoholic infusion, decoction	Oral ingestion	Back symptom/complaint, Flank/axilla symptom/complaint, Muscle pain	[2,6,12]
Tetrastigma cruciatum Craib and Gagnep.	VITACEAE	1	Roots	Decoction	Oral ingestion	Muscle pain	[6]
Thunbergia coccinea Wall. ex D. Don	ACANTHACEAE	2	Stems, whole plants	Decoction	Oral ingestion	Arm symptom/complaint, Knee symptom/complaint, Muscle pain	[2,6]
Thunbergia laurifolia Lindl.	ACANTHACEAE	3	Leaves, roots, stems, whole plants	Decoction	Oral ingestion	Muscle pain	[2,5,6]
Thysanolaena latifolia (Roxb. ex Hornem.) Honda	POACEAE	1	Roots	Decoction	Oral ingestion	Muscle pain	[2]

Tinospora crispa (L.) Hook.f. and Thomson	MENISPERMACEAE	4	Aerial parts, stems	Alcoholic infusion, sugar infusion, decoction	Oral ingestion, poultices	Back symptom/complaint, Flank/axilla symptom/complaint, Muscle pain	[2,6,7,11]
Tupistra muricata (Gagnep.) N. Tanaka	ASPARAGACEAE	2	Leaves, roots	Burning, decoction	Poultices	Fracture: femur, Leg/thigh symptom/complaint, Sprain/strain of joint NOS, Wrist symptom/complaint	[2,6]
Turpinia pomifera (Roxb.) DC.	STAPHYLEACEAE	1	Roots	Alcoholic infusion, decoction	Oral ingestion	Muscle pain	[6]
Uncaria laevigata Wall. ex G. Don	RUBIACEAE	1	Roots	Decoction	Oral ingestion	Muscle pain	[2]
Xantolis burmanica (Collett and Hemsl.) P. Royen	SAPOTACEAE	1	Bark	Decoction	Oral ingestion	Muscle pain	[6]
Zingiber latifolium Theilade and Mood	ZINGIBERACEAE	1	Roots	Decoction	Oral ingestion	Fracture: other	[3]
Zingiber officinale Roscoe	ZINGIBERACEAE	1	Roots	Grind	Oral ingestion	Muscle pain	[5]
Zingiber ottensii Valeton	ZINGIBERACEAE	2	Roots	Decoction	Oral ingestion	Back symptom/complaint, Flank/axilla symptom/complaint, Muscle pain	[6,7]

Zingiber purpureum Roscoe	ZINGIBERACEAE	2	Roots	Concoction, grind	Poultices	Muscle pain, Sprain/strain of joint NOS	[8,10]
Ziziphus cambodianus Pierre	RHAMNACEAE	2	Barks	Decoction	Oral ingestion	Muscle pain	[1,5]

References

1. Kamwong, K. Ethnobotany of Karens at Ban Mai Sawan and Ban Huay Pu Ling, Ban Luang Sub-District, Chom Thong District, Chiang Mai Province. Master's Thesis, Chiang Mai University, Chiang Mai, Thailand, 2009.
2. Kaewsangsai, S. Ethnobotany of Karen in the Royal Project Extended Area Khun Tuen Noi village, Omkoi district, Chiang Mai province. Master's Thesis, Chiang Mai University, Chiang Mai, Thailand, 2017.
3. Sukkho, T. A Survey of Medicinal Plants Used by Karen People at Ban Chan and Chaem Luang Subdistricts, Mae Chaem District, Master's Thesis, Chiang Mai Province. Chiang Mai University, Chiang Mai, Thailand, 2008.
4. Junsongduang, A. Roles and Importance of Sacred Forest in Biodiversity Conservation in Mae Chaem District, Chiang Mai Province. Doctor of Philosophy, Chiang Mai University, Chiang Mai, Thailand, 2013.
5. Winjchiyanan, P. Ethnobotany of Karen in Chiang Mai. Master's Thesis, Chiang Mai University, Chiang Mai, Thailand, 1995.
6. Kantasrila, R. Ehtnobotany of Karen at Ban Wa Do Kro, Mae Song Sub-district, Tha Song Yang District, Tak Province. Master's Thesis, Chiang Mai University, Chiang Mai, Thailand, 2016.
7. Tangjitman, K. Vulnerability prediction of medicinal plants used by Karen people in Chiang Mai province to climatic change using species distribution model (SDM). Doctor of Philosophy, Chiang Mai University, Chiang Mai, Thailand, 2014.
8. Junkhonkaen, J. Ethnobotany of Ban Bowee, Amphoe Suan Phueng, Changwat Ratchaburi. Master's Thesis, Kasetsart University, Bankok, Thailand, 2012.
9. Pongamornkul, W. An Ethnobotanical Study of the Karen at Ban Yang Pu Toh and Ban Yang Thung Pong, Chiang Dao District, Chiang Mai Province. Master's Thesis, Chiang Mai University, Chiang Mai, Thailand, 2003.
10. Sonsupub, B. Ethnobotany of Karen Community in Raipa Village, Huaykhayeng Subdistrict, Thongphaphume District, Kanchanaburi Province. Master's Thesis, Kasetsart University, Bankok, Thailand, 2010.
11. Tangjitman, K. Ethnobotany of the Karen at Huay Nam Nak village, Tanaosri subdistrict, Suanphueng district, Ratchaburi province. *Thai J. Bot.* **2017**, *9(2)*, 253-272.
12. Trisonthi, C.; Trisonthi, P. Ethnobotanical study in Thailand, a case study in Khun Yuam district Maehongson province. *Thai J Bot* **2009**, *1*, 1-23.
13. Mahawongsanan, A. Change of Herbal Plants Utilization of the Pga K'nyau: A Case study of Ban Huay Som Poy, Mae Tia Watershed, Chom Thong District, Master's Thesis, Chiang Mai Province. Chiang Mai University, Chiang Mai, Thailand, 2008.
14. Puling, W. Ethnobotany of Karen for studying medicinal plants at Angka Noi and Mae Klangluang villages, Chomthong district, Chiang Mai. B.Sc. Special issue, Chiang Mai University, Chiang Mai, Thailand, 2001.
15. Moonjai, J. Ethnobotany of Ethnic Group in Mae La Noi District, Mae Hong Son Province. Master's Thesis, Chiang Mai University, Chiang Mai, Thailand, 2017.
